# Relative sorption coefficient: Key to tracing petroleum migration and other subsurface fluids

**DOI:** 10.1038/s41598-019-52259-6

**Published:** 2019-11-14

**Authors:** L. Zhang, Y. Wang, M.-W. Li, Q.-Z. Yin, W. Zhang

**Affiliations:** 1 Key Laboratory of Petroleum Resources Research, Institute of Geology and Geophysics, Chinese Academy of Sciences, Beijing, 100029 China; 2 Innovation Academy for Earth Science, Chinese Academy of Sciences, Beijing, 100029 China; 3 Department of Earth, Ocean and Atmospheric Science, Florida State University, and National High Magnetic Field Laboratory, Tallahassee, FL 32306-4100 USA; 4 Institute of Groundwater and Earth Sciences, Jinan University, Guangzhou, P.R. China; 5 Sinopec Key Laboratory of Petroleum Accumulation Mechanisms, Sinopec Research Institute of Petroleum Exploration and Production, 31 Xueyuan Road, Beijing, 100083 China; 6 Department of Earth and Planetary Sciences, University of California at Davis, One Shields Avenue, Davis, CA 95616 USA; 7 Changqing Oilfield Company, PetroChina, Xi’an, Shanxi 710021 China

**Keywords:** Geochemistry, Economic geology, Crude oil

## Abstract

The accumulation and spatial distribution of economically important petroleum in sedimentary basins are primarily controlled by its migration from source rocks through permeable carrier beds to reservoirs. Tracing petroleum migration entails the use of molecular indices established according to sorption capacities of polar molecules in migrating petroleum. However, little is known about molecular sorption capacities in natural migration systems, rendering these indices unreliable. Here, we present a new approach based on a novel concept of relative sorption coefficient for quantitatively assessing sorption capacities of polar molecules during natural petroleum migration. Using this approach, we discovered previously unrecognized “stripping” and “impeding” effects that significantly reduce the sorption capacities of polar compounds. These discoveries provide new insights into the behaviors of polar compounds and can easily explain why traditional molecular indices yield incorrect information about petroleum migration. In light of these new findings, we established new molecular indices for tracing petroleum migration. We demonstrate via case studies that the newly established indices, unlike traditional molecular indices, are reliable and effective in tracing petroleum migration. Our approach can be applied to diverse basins around the world to reveal distribution patterns of petroleum, which would decrease environmental risks of exploration by reducing unsuccessful wells.

## Introduction

**Figure 1:**
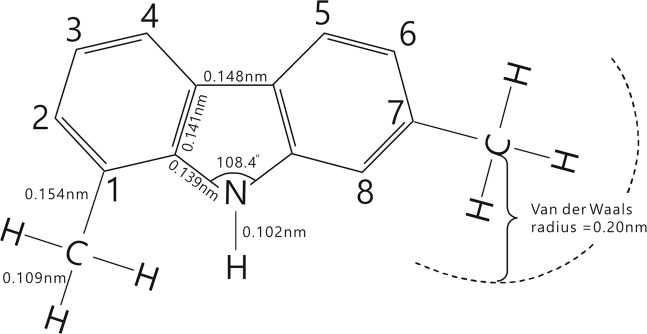
Structure of 1,7-dimethylcarbazole. The bond length data and angles are from Kurahashi *et al*.^32^ and Song^33^; the Van der Waals radii of a methyl are from Chen *et al*.^34^.

Petroleum is produced in source rocks through thermal alteration of organic matter buried in sedimentary basins. Its accumulation and distribution are mainly controlled by secondary petroleum migration (SPM) through permeable carrier beds following petroleum expulsion (primary migration) out of the source rocks. Tracing petroleum migration can reveal distribution patterns of petroleum reservoirs and thus increase exploration success rate. Meanwhile, environmental risks can be decreased by reducing unsuccessful wells. Biomarker hydrocarbon geochemistry can be applied to trace SPM^1–6^. The underlying principle of this approach is that polar molecules are preferentially removed from petroleum during secondary migration due to sorption onto immobile mineral surfaces, which causes their concentrations to decrease with increasing migration distance^1,3,5,7^. Thus, tracing SPM entails the use of molecular indices established on the basis of sorption capacities of polar compounds in migrating petroleum (Supplementary Text S-1.1). However, there have been no reports of quantitative research on the sorption capacities of polar molecules in naturally migrating petroleum, although it has been suggested that the sorption capacities of alkylcarbazoles are determined by the (partial) shielding effect related to alkylation at positions 1 and/or 8 (refs 3, 6) (Fig. 1). Sorption of trace polar compounds during lateral petroleum migration typically reaches equilibrium^2,4,5,8^ and thus their sorption capacities essentially represent equilibrium sorption capacities determined by both sorption and desorption. However, the shielding and partial shielding effects consider only the sorption, not the desorption, of alkylcarbazoles during petroleum migration. Therefore, the previously-proposed theory on molecular sorption capacities, which was based solely on the shielding and partial shielding effects, needs to be re-evaluated to avoid erroneous results when applying molecular indices to trace SPM, as demonstrated by the case studies herein.

In this paper, we propose a new parameter, the Relative Sorption Coefficient (RSC), which quantitatively describes equilibrium sorption capacities of polar compounds in migrating petroleum. We establish its computation method (see Methods), and then apply and test the validity of the new approach, by using natural petroleum samples collected from the Xifeng Oilfield in the Ordos Basin and the Rimbey-Meadowbrook Reef Trend in the Western Canada Sedimentary Basin (WCSB). Because the petroleum in the Xifeng Oilfield contains very little benzocarbazoles^5^, we only analyzed alkylcarbazoles in the samples from this oilfield, although both alkylcarbazoles and benzocarbazoles can serve as important tracers^1,5,9,10^. Using the RCS approach, we determined the sorption capacities of an important group of polar molecules, the alkylcarbazoles, and discovered two previously unrecognized effects (i.e., stripping and impeding effects) that strongly influence the equilibrium sorption capacities of polar compounds. In light of these new findings, we reclassify alkylcarbazoles into six subgroups, according to their equilibrium sorption capacities, and propose new ratios that consist of numerators with stronger sorption capacities than denominators. All the new ratios show a significantly decreasing trend with increasing migration distance, demonstrating that the new ratios are reliable and effective indices for migration and that the RSC provides the key to tracing SPM. Our new method is further validated through the analyses of benzocarbazoles in petroleum samples from the Rimbey-Meadowbrook reef trend in the WCSB. Because the RSC is established on the basis of a physicochemical sorption model, it should be widely applicable to assessing equilibrium sorption capacities of solutes in a diverse range of geofluids.

## Results

### Modification of secondary migration fractionation index

We have previously investigated SPM in the Xifeng Oilfield in the southwest part of the Ordos Basin in China using the secondary migration fractionation index (*SMFIs*). The geological setting, samples and geochemical data are documented in Zhang *et al*.^5^. Besides sorption, this earlier study also examined other factors^11–20^ that may influence the concentrations of polar compounds in migrating petroleum. It illustrated that the effect of thermal maturation of source rocks on polar molecule can be eliminated in the derivation of *SMFIs* (see Zhang *et al*.^5^ for details). Other influences (i.e., organic facies of source rocks, biodegradation of petroleum and dissolution in water) can be neglected for alkylcarbazoles in the petroleum of this field^5^. In this study, we re-calculated the *SMFI*s, using the migration-sorption fractionation equation with a quadratic polynomial (Supplementary Equation (S3)), to improve the accuracy of the results (Supplementary Text, S-1.2-3). The results clearly show an exponential decrease of *SMFI*s with increasing relative migration distance (Supplementary Fig. S1A–C,G–I), suggesting that the Xifeng Oilfield was likely formed by SPM in the SW direction along the sand body from the source kitchen located in the NE of the reservoir (refer to Zhang *et al*.^5^).

**Figure 2:**
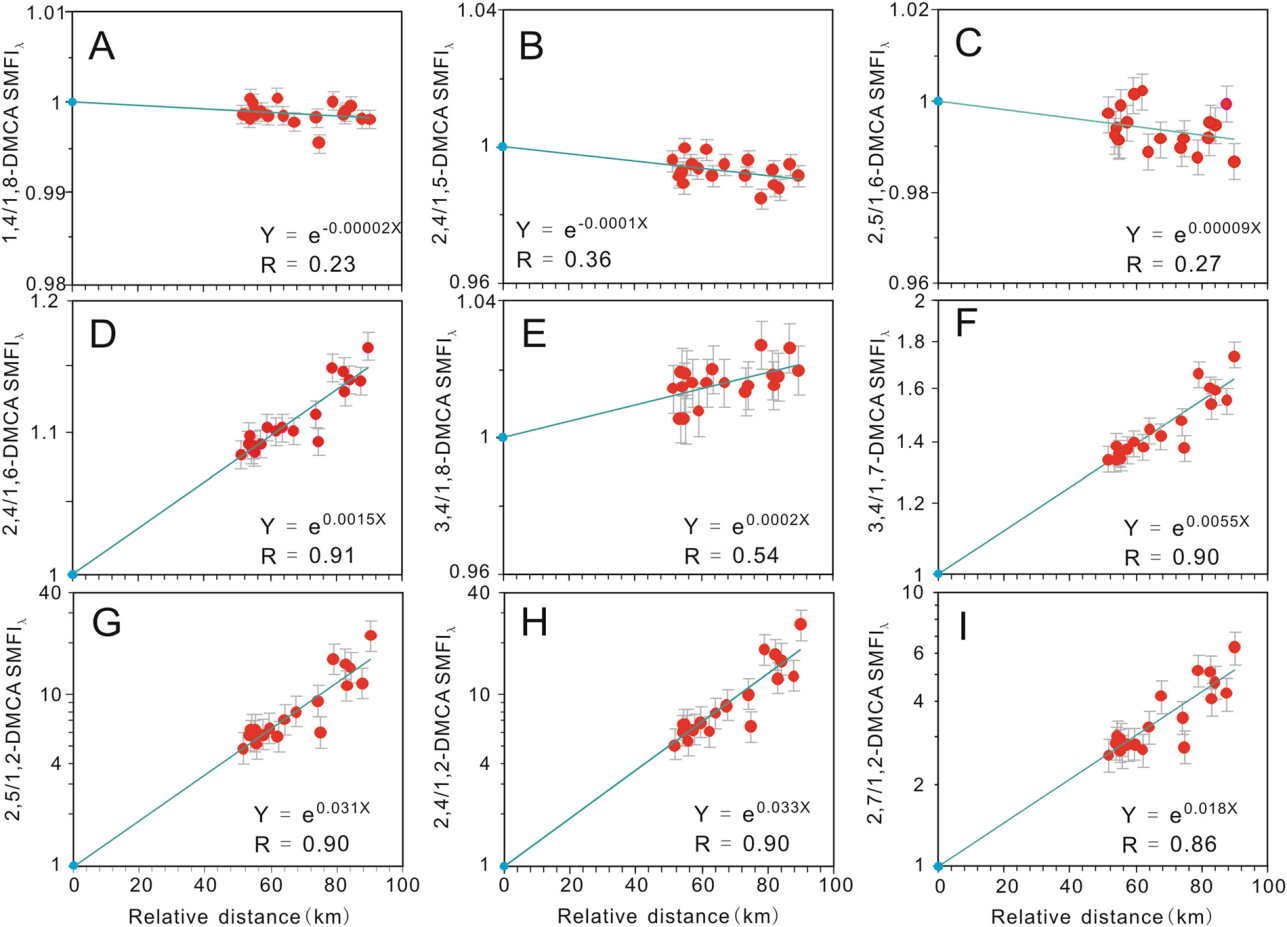
Correlation diagrams showing the relationships between relative migration distances and the *SMFI*_*λ*_ ratios of different groups of dimethylcarbazoles in the Xifeng Oilfield. *SMFI*_*λ*_: the amended Secondary Migration Fractionation Index; DMCA: dimethylcarbazole. The number of data points in each panel is nineteen; the grey error bars indicate one standard deviation (1σ) from the logarithmic values of the *SMFI*_*λ*_ ratios. 1,4/1,8-DMCA *SMFI*_*λ*_: ratio of the *SMFI*_*λ*_ of 1,4-DMCA to the *SMFI*_*λ*_ of 1,8-DMCA **(A)**; 3,4/1,8-DMCA *SMFI*_*λ*_: ratio of the *SMFI*_*λ*_ of 3,4-DMCA to the *SMFI*_*λ*_ of 1,8-DMCA (**B**); 2,4/1,5-DMCA *SMFI*_*λ*_: ratio of the *SMFI*_*λ*_ of 2,4-DMCA to the *SMFI*_*λ*_ of 1,5-DMCA (**C**); 2,4/1,6-DMCA *SMFI*_*λ*_: ratio of the *SMFI*_*λ*_ of 2,4-DMCA to the *SMFI*_*λ*_ of 1,6-DMCA (**D**); 2,5/1,7-DMCA *SMFI*_*λ*_: ratio of the *SMFI*_*λ*_ of 2,5-DMCA to the *SMFI*_*λ*_ of 1,7-DMCA (**E**); 2,7/1,2-DMCA *SMFI*_*λ*_: ratio of the *SMFI*_*λ*_ of 2,7-DMCA to the *SMFI*_*λ*_ of 1,2-DMCA (**F**); 3,4/1,7-DMCA *SMFI*_*λ*_: ratio of the *SMFI*_*λ*_ of 3,4-DMCA to the *SMFI*_*λ*_ of 1,7-DMCA (**G**); 2,5/1,2-DMCA *SMFI*_*λ*_: ratio of the *SMFI*_*λ*_ of 2,5-DMCA to the *SMFI*_*λ*_ of 1,2-DMCA (**H**); 2,4/1,2-DMCA *SMFI*_*λ*_: ratio of the *SMFI*_*λ*_ of 2,4-DMCA to the *SMFI*_*λ*_ of 1,2-DMCA (**I**). The *SMFI*_*λ*_ ratio of 1 at the reference point (x = 0 km) is the model value, and was excluded from regression analyses shown in this figure.

However, the information about petroleum migration derived from the *SMFI*s needs to be verified by using the ratios of the *SMFI*s. Because the *SMFI* is affected by the relative rates of concentration variations of polar molecules at the starting point or at a reference point of SPM (Supplementary Text, S-1.2-3), it cannot be used to construct reliable ratios. To overcome this problem, we revised the *SMFI* (Supplementary Equation (S19)) and its related ratios (Supplementary Text, S-1-3). The amended indices are denoted by the subscript λ (e.g. *SMFI*_*λ*_). The values of *SMFI*_*λ*_ (Supplementary Table S4) display similar distribution trends as the values of *SMFI* Supplementary Fig. S1), even though the powers of their regression equations are different, as shown by Supplementary Equation (S16). The ratios of the *SMFI*_*λ*_s of alkylcarbazoles with stronger sorption capacities to those with weaker sorption capacities should decrease with increasing migration distance if the underlying assumptions about source facies, biodegradation and thermal maturity effects are valid. The ratios were initially established based on the previously proposed theory about sorption capacities that considered only the shielding and partial shielding effects^3,6,21,22^. Based on this theory, alkylcarbazole isomers can be divided into three groups^3,6^: N-H shielded (Group I), N-H partially shielded (Group II) and N-H exposed (Group III). Their sorption capacities are expected to decrease in the order of Group III> Group II> Group I, and the ratios of the *SMFI*_*λ*_*s* of dimethylcarbazoles (DMCAs) in Group III to those in Group II, III to I and II to I would be predicted to decrease with increasing migration distance. However, many of these ratios (Fig. 2) do not display a decreasing trend with increasing migration distance, but instead they exhibit a clear increasing trend (Supplementary Text, S-1.5), which is completely opposite to the decreasing trend of *SMFIs* and *SMFI*_*λ*_s (Supplementary Fig. S1), and inconsistent with the geological conditions (Fig. 1 in Zhang *et al*.^5^). From this, we can see that if the ratios of carbazoles, constructed on the basis of the existing theory on sorption capacities, are used to trace SPM, it would yield erroneous or misleading information about petroleum migration. Similarly, the ratios based on the current sorption capacity theory cannot be used to verify the information about SPM that is inferred from the *SMFI*s and *SMFI*_*λ*_s. Therefore, their use should be discontinued.

### Relative sorption coefficient (RSC)

We re-examined the sorption capacities of polar compounds in petroleum samples from the Xifeng Oilfield using our new approach described in the Methods and Supplementary Text S-1. We calculated the relative sorption coefficients - the *K*_*r*_ values of alkylcarbazoles in these petroleum samples (Supplementary Table S2). The *K*_*r*_ values vary widely. Some N-H partially shielded DMCAs (Group II) have higher *K*_*r*_ values than some of the N-H exposed DMCAs (Group III), which cannot be explained by the existing theory on sorption capacities that considered only the shielding and partial shielding effects. This suggests that there are other factors controlling equilibrium sorption capacities of alkylcarbazoles.

Through comparison of desorption of the adsorbed polar compounds under both static and dynamic conditions (Supplementary Text, S-1.6), a stripping effect was observed arising from petroleum migration that causes “tall” alkylcarbazoles to desorb more easily than the “short” ones (Supplementary Figs S4, S5). This stripping effect greatly reduces the equilibrium sorption capacities of alkylcarbazoles with the alkyl substituents at positions 4 and/or 5, as is demonstrated by the *K*_*r*_ values.

**Figure 3:**
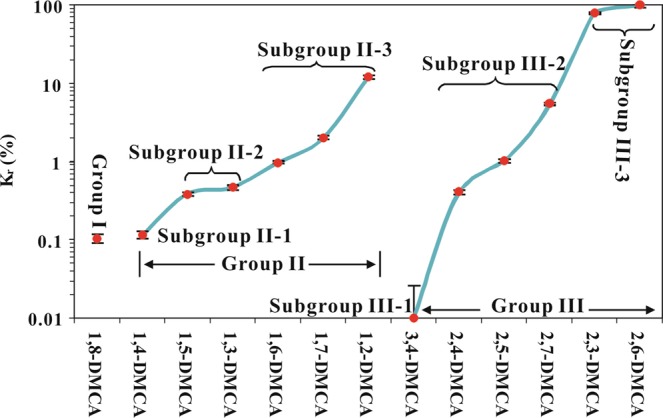
The sequence of individual dimethylcarbazoles in different groups sorted by their relative sorption coefficients. DMCA = dimethylcarbazole; *K*_*r*_ = relative sorption coefficient (%). In order to plot the datum point of 3,4-DMCA (Kr = 0%) using the logarithmic coordinate, 0.01% were added to all the *K*_*r*_ values. The black bars indicate the errors of the relative sorption coefficient calculated with Supplementary Equation (S29) in Supplementary Text S-1.4.

In Group II alkylcarbazoles, the molecular height of 1,4-DMCA is greater than for 1,5- and 1,3-DMCA (Supplementary Text, S-1.6), and the latter two are taller than the other DMCAs in this group. Consequently, 1,4-DMCA has a lower *K*_*r*_ value than 1,5- and 1,3-DMCA, which have lower *K*_*r*_ values than the rest of the DMCAs in this group (Supplementary Fig. S3). In light of the stripping effect, the Group II alkylcarbazoles are further divided into three subgroups (Fig. 3) with decreasing stripping effect and increasing sorption capacity in the following order: N-H partially shielded alkylcarbazole with the alkyl at position 4 (Subgroup II-1), N-H partially shielded alkylcarbazoles with the alkyl at positions 3 or 5 (Subgroup II-2), and N-H partially shielded without the alkyl at position 3, 4 or 5 (Subgroup II-3) (Figs 3 and S3).

In Group III alkylcarbazoles, 3,4-DMCA has two methyls sticking out, and is subject to a stronger stripping effect (two-methyl stripping; see Supplementary Text, S-1.6 for details) and thus has a lower *K*_*r*_ value than 2,4- or 2,5-DMCA (Supplementary Fig. S5A–C). The *K*_*r*_ value of 3,4-DMCA is even lower than that of 1,8-DMCA, which experiences the shielding effect (Fig. 3).

In addition, we discovered an impeding effect related to the alkyls at positions 2 and 7 (Supplementary Text, S-1.7). The impeding effect causes the equilibrium sorption capacity of 2,7-DMCA in the Group III alkylcarbazoles to become lower than those of 2,3- and 2,6-DMCA (Supplementary Figs. S5D–F). Due to the stripping and impeding effects, alkylcarbazoles in Group III show large variations in *K*_*r*_ (Supplementary Fig. S4) and are also divided into three subgroups (Fig. 3): N-H exposed alkylcarbazole with the alkyls at positions 3 and 4 (Subgroup III-1), N-H exposed alkylcarbazoles with one alkyl at position 2 and the other alkyl at positions 4, 5 or 7 (Subgroup III-2), and N-H exposed alkylcarbazoles without the alkyls at positions 4, 5 or 7 (Subgroup III-3).

The stripping and impeding effects, which control the sorption capacities of polar molecules in migrating petroleum, are also related to the molecular structures of the organic compounds, just like the shielding effect. Based on the three effects noted above and the *K*_*r*_ values (Supplementary Table S2), these subgroups and the Group I alkylcarbazole can be arranged in the following sequence with decreasing equilibrium sorption capacity (Fig. 3): Subgroup III-3 (*K*_*r*_ = 78.5–100%)> III-2 (*K*_*r*_ = 0.40–5.6%), II-2 and II-3 [II-3 (*K*_*r*_ = 0.97–12.0%)> II-2 (*K*_*r*_ = 0.38–0.46%)]> II-1 (*K*_*r*_ = 0.11%) and Group I (*K*_*r*_ = 0.10%)> III-1 (*K*_*r*_ = 0.0%).

From the above analyses of molecular structures and their relationships with *K*_*r*_, we established the following sequence of various effects on reducing the equilibrium sorption capacities: two-methyl stripping (represented by Subgroup III-1)> shielding (Group I)> partial shielding plus one-methyl stripping (II-1 and -2)> partial shielding (II-3), one-methyl stripping and impeding (III-2)> partial impeding. The interplay of these three effects results in complex variations in equilibrium sorption capacities of the DMCAs within and among subgroups. The seemingly unreasonable relationships of *SMFI* ratios with relative migration distance (Fig. 2) can all be explained by these effects and their combination(Supplementary Text S-1.8).

The relative sorption coefficient is derived from the linear isotherm model that is the simplification of the Langmuir isotherm model of equilibrium sorption at low concentrations of adsorbents such as carbazoles (refer to the Methods Section, Supplementary Information and Zhang *et al*.^5^). Recent studies on sorption of asphaltenes onto minerals show that the Langmuir isotherm model can be used to describe the equilibrium adsorption of asphaltenes when interactions between the solute and the solvent as well as interactions that can occur at a non-ideal lattice of a mineral are negligible and that the sorption of asphaltenes is highly dependent on the heteroatoms (i.e. N, O, S) in their molecular structure^23–25^. These results confirm the validity of using the linear isotherm model to investigate the equilibrium sorption of polar heteroatom compounds such as carbazoles onto solid surfaces.

### New ratios and their application

Given the sorption capacity sequence of the subgroups and Group I, eighteen *SMFI*_*λ*_ ratios are established as indices for petroleum migration: alkylcarbazoles in Subgroup III-3 to those in III-2, III-3 to III-1, III-3 to II-3, III-3 to II-2, III-3 to II-1, III-3 to Group I, III-2 to III-1, III-2 to II-1, III-2 to Group I, II-3 to II-2, II-3 to II-1, II-3 to Group I, II-3 to III-1, II-2 to II-1, II-2 to Group I, II-2 to III-1, II-1 to III-1, and Group I to III-1. Since the equilibrium sorption capacities of the numerators are significantly higher than those of the denominators, these ratios decrease with increasing migration distance and thus can serve as odometers for SPM (Supplementary Equation (S23)). Similarly, the corresponding ratios of the geometric means of *SMFI*_*λ*_s decrease with increasing migration distance and can also be used as indices for petroleum migration (Supplementary Equations (S21 and S22)). It is worth noting that the ratios of alkylcarbazoles within each group (except Group I with only one compound), which were not considered previously, can also be useful in the establishment of new indices (Supplementary Text S-1.9).

**Figure 4:**
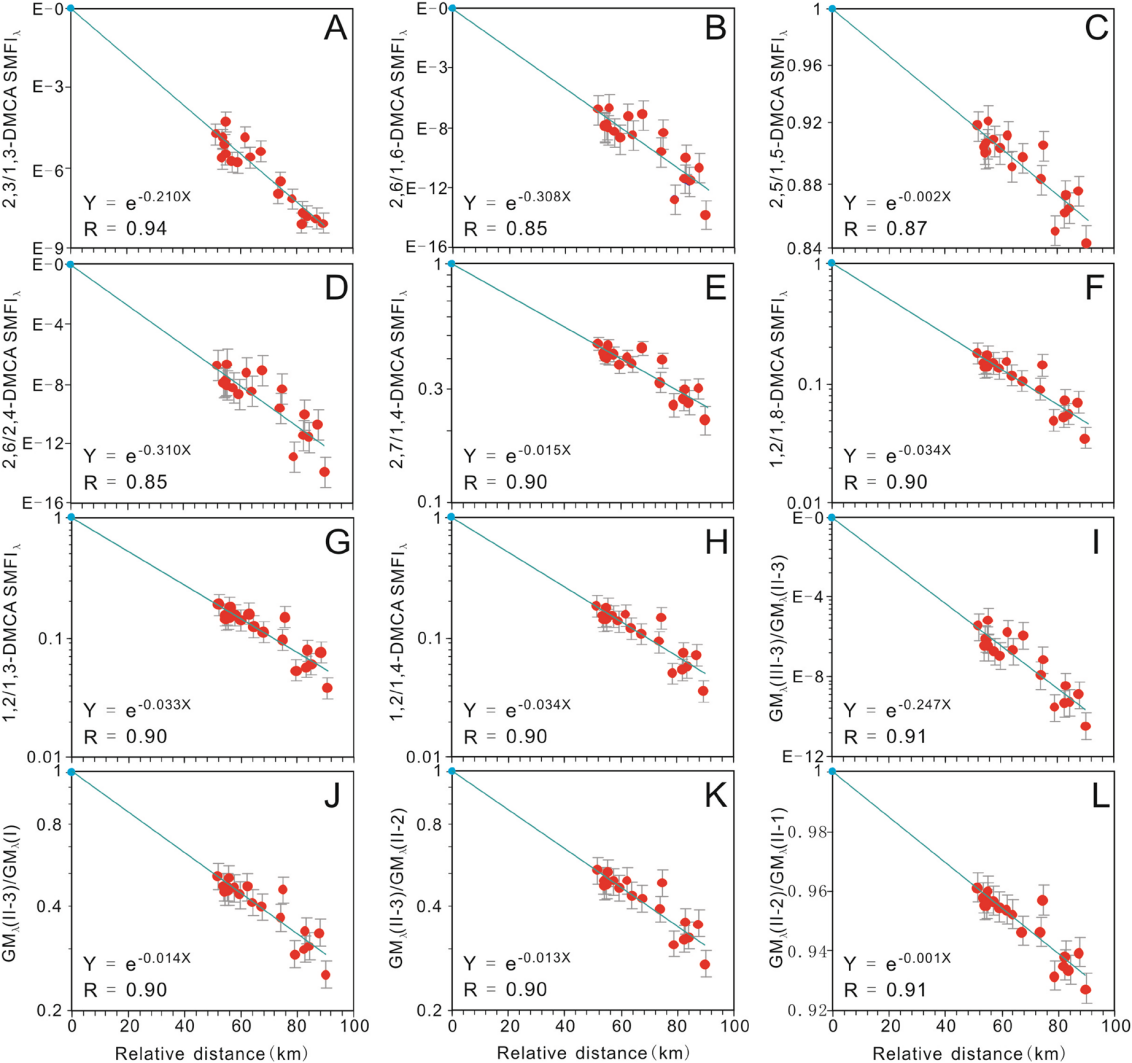
Correlation diagrams showing the relationships between relative migration distances and *SMFI*_*λ*_ ratios of different subgroups of dimethylcarbazoles in the Xifeng Oilfield. *SMFI*_*λ*_: the modified Secondary Migration Fractionation Index; DMCA: dimethylcarbazole. The number of the data points in each panel is nineteen; the grey error bars indicate 1σ from the logarithmic values of the *SMFI*_*λ*_ ratios. 2,3/1,3-DMCA *SMFI*_*λ*_: ratio of the *SMFI*_*λ*_ of 2,3-DMCA to the *SMFI*_*λ*_ of 1,3-DMCA (**A**); 2,6/1,6-DMCA *SMFI*_*λ*_: ratio of the *SMFI*_*λ*_ of 2,6-DMCA to the *SMFI*_*λ*_ of 1,6-DMCA (**B**); 2,5/1,5-DMCA *SMFI*_*λ*_: ratio of the *SMFI*_*λ*_ of 2,5-DMCA to the *SMFI*_*λ*_ of 1,5-DMCA (**C**); 2,6/2,4-DMCA *SMFI*_*λ*_: ratio of the *SMFI*_*λ*_ of 2,6-DMCA to the *SMFI*_*λ*_ of 2,4-DMCA (**D**); 2,7/1,4-DMCA *SMFI*_*λ*_: ratio of the *SMFI*_*λ*_ of 2,7-DMCA to the *SMFI*_*λ*_ of 1,4-DMCA (**E**); 1,2/1,8-DMCA *SMFI*_*λ*_: ratio of the *SMFI*_*λ*_ of 1,2-DMCA to the *SMFI*_*λ*_ of 1,8-DMCA (**F**); 1,2/1,3-DMCA *SMFI*_*λ*_: ratio of the *SMFI*_*λ*_ of 1,2-DMCA to the *SMFI*_*λ*_ of 1,3-DMCA (**G**); 1,2/1,4-DMCA *SMFI*_*λ*_: ratio of the *SMFI*_*λ*_ of 1,2-DMCA to the *SMFI*_*λ*_ of 1,4-DMCA (**H**). GM_λ_(III-3), GM_λ_(II-3) and GM_λ_(II-2): the geometric means of the *SMFI*_*λ*_*s* of subgroups III-3, II-3, II-2 and II-1, respectively. GM_λ_(I) and GM_λ_(II-1) are the *SMFI*_*λ*_*s* of 1,8-DMCA and 1,4-DMCA, respectively (refer to Fig. 3). The *SMFI*_*λ*_ ratio of 1 at the reference point (x = 0 km) is the model value, which was excluded in the regression analyses in this figure.

The new *SMFI*_*λ*_ ratios for the Xifeng Oilfield fit the known data well, clearly showing exponential decreases with increasing migration distance with high correlation coefficients (Fig. 4). These are consistent with the migration fractionations inferred from *SMFI*_*λ*_s and *SMFIs*, and geological conditions^26–29^ (Fig. 1 in Zhang *et al*.^5^). Thus, the new ratios confirm the validity of the influence elimination and migration information revealed by the *SMFI*s^5^ and *SMFI*_*λ*_s, and demonstrate that the petroleum migrated along the sand body from the source kitchen into the Xifeng Oilfield in a SW direction (refer to Zhang *et al*.^5^).

Molecular indices that are correlated with migration directions, pathways and distances have been sought based on sorption capacities of polar organic compounds in migrating petroleum for decades^1,5^, but with limited success^5^ because the sorption capacities of these polar compounds have been unclear. As a result, reliable indices have not been established and secondary petroleum migration still remains the least understood of the processes involved in petroleum accumulation^5^. The results of the application of the new ratios of alkylcarbazoles in the petroleum of the Xifeng Oilfield, however, demonstrate that the relative sorption coefficients (*K*_*r*_) can be used to assess the sorption capacities and that the new ratios established on the basis of the new understanding of the sorption capacities can serve as effective indices for petroleum migration. These new indices provide a powerful tool for revealing migration directions, pathways and distances that control petroleum distribution patterns in reservoirs in basins, which would greatly facilitate future petroleum exploration and increase the success rate of wells.

Furthermore, the new observation of the stripping effect on equilibrium sorption capacities is supported by the analyses of benzocarbazoles in the petroleum samples from the Rimbey-Meadowbrook reef trend of central Alberta, Canada. In these petroleum samples, the “taller” benzo[c]carbazole has a lower *K*_*r*_ value than the “shorter” benzo[a]carbazole, consistent with predictions from the stripping effect (Supplementary Table S5).

## Discussion

The new concept of RSC overcomes the dependency of the sorption coefficient assessment on the migration velocity and rock characteristics of the carrier beds. As demonstrated in our case studies, the RSC provides a powerful tool with a sound scientific basis to quantitatively evaluate equilibrium sorption capacities of polar compounds during petroleum migration, and can help uncover factors controlling equilibrium sorption capacities. Without this tool, it would be impossible to quantitatively assess equilibrium sorption capacities of polar compounds in migrating petroleum and to establish reliable molecular indices for tracing petroleum migration. The lack of a quantitative assessment tool is also the primary reason why many of the previously proposed molecular indices failed to provide reliable information about SPM. Application of this approach to quantitative assessment of equilibrium sorption capacities of alkylcarbazoles has resulted in the discovery of the previously unrecognized stripping and impeding effects that significantly reduce the equilibrium sorption capacities of polar compounds. These findings have led to the reclassification of the polar compounds according to their sorption capacities. Based on the reclassification of the polar compounds, we established eighteen new ratios. As demonstrated in our case studies, these new indices provide reliable information about petroleum migration (i.e. migration directions, routes and distances). Therefore, this approach is the key to tracing secondary petroleum migration and can be applied to petroliferous basins around the world, to reveal distribution patterns of petroleum reservoirs, which would help to find more petroleum and decrease environmental risks of exploration by reducing unsuccessful wells.

Moreover, the concept of RSC and its evaluation method developed in this study should be applicable in hydrological and environmental studies (as well as other disciplines) to trace the movement of pollutants and water (and other geofluids) (Supplementary Text S-1.10).

## Methods

The equilibrium sorption of a polar molecule or an adsorbable element in a natural migration system of petroleum or other geofluids can be described by the linear isotherm model if its concentration is sufficiently low^5,8,30,31^. In this physicochemical model, the sorption coefficient *K*_*d*_ (cm^3^/g) represents the sorption amount of a polar compound or an adsorbable element at a given concentration and saturation of petroleum or geofluid^8,30,31^ (see Supplementary Equation (S5) in Zhang *et al*.)^5^. This amount may describe the equilibrium sorption capacity of the compound or element, according to Delle Site^8^. However, the *K*_*d*_ values determined in laboratories are not necessarily applicable to natural migration systems, due to differences in size, time and distance between laboratory experiments and natural migration systems. Moreover, lab experimental studies for the determination of sorption coefficients are expensive and time consuming, and the results may not be accurate, especially when concentrations are low^8^. Above all, *K*_*d*_ is also controlled by many factors such as the porosity, density of carrier beds and the average velocity of migration (Supplementary Text S-1.2). Therefore, the sorption coefficient *K*_*d*_ cannot be used directly to describe the equilibrium sorption capacities of polar organic compounds or trace elements during lateral migration.

To evaluate equilibrium sorption capacities of polar compounds (or adsorbable elements) in natural migration systems, we introduce a new concept of relative sorption coefficient (RSC):

1 $${K}_{r}=\frac{{K}_{d}-{K}_{dmin}}{{K}_{dmax}-{K}_{dmin}}\times 100( \% )$$

where *K*_*r*_ is the RSC; *K*_*d*_ is the sorption coefficient (cm^3^/g); *K*_*dmax*_ is the maximum value in a series of *K*_*d*_ values of polar compounds in petroleum (or adsorbable elements in other geofluids); and *K*_*dmin*_ is the minimum value. The range of *K*_*r*_ values is 0–100%. *K*_*r*_ can be used quantitatively to evaluate equilibrium sorption capacities. High *K*_*r*_ values indicate strong equilibrium sorption capacities.

For the linear isotherm model of the equilibrium sorption in natural migration systems^5,8,30,31^, we can derive the following equation from Supplementary Equation (S8) in Zhang *et al*.^5^:

2 $${K}_{d}=({R}_{d}-1)\frac{n}{{n}_{s}\cdot {\rho }_{s}}\,$$

where *R*_*d*_ represents the retardation factor of a polar compound in migrating petroleum or an adsorbable trace element in migrating groundwater (a dimensionless constant), being related to the sorption of the compound or the element and the average migrating velocity of petroleum or groundwater (Supplementary Text S-1.2); *n* is the porosity of the carrier bed (%); *n*_*s*_ = *100 − n* (%); *ρ*_*s*_ is the density of the solids (g/cm^3^).

Migration of petroleum (or other geofluids) usually occurred in past geological times. Therefore, the current porosity and density of carrier beds do not represent the porosity and density during migration, as these lithological properties most likely have changed over time during diagenesis. Therefore, quantitative measurements of the porosity and density of carrier beds during migration can rarely be obtained. However, these parameters are the same for different compounds or for different elements in a migration system, and thus can be eliminated (Supplementary Text S-1.2) when Eq. (2) is substituted into Eq. (1):

3 $${K}_{r}=\frac{{R}_{d}-{R}_{dmin}}{{R}_{dmax}-{R}_{dmin}}\times 100( \% )$$

where *R*_*dmax*_ is the maximum value in a series of *R*_*d*_ values of polar compounds or elements; *R*_*dmin*_ is the minimum value. *R*_*d*_ is also controlled by the average velocity of migration and the difference in relative variation rates of concentrations with time at the starting point of a migration pathway between polar compounds. However, it is demonstrated that the RSC can also eliminate these two kinds of influences when Supplementary Equations (S11–S13) are substituted into Eq. (3) (see Supplementary Text S-1.2 for details):

4 $${K}_{r}=\frac{{a}_{\lambda max}-{a}_{\lambda }}{{a}_{\lambda max}-{a}_{\lambda min}}\times 100( \% )$$

where ${a}_{\lambda }$ is a constant controlling migration-sorption fractionation (km^−1^) and can be derived from Supplementary Equation (S9); ${a}_{\lambda max}$is the maximum in a series of ${a}_{\lambda }$ values of polar compounds (km^−1^); ${a}_{\lambda min}$ is the minimum (km^−1^). Equation (4) provides a workable means to quantitatively evaluate sorption capacities of polar organic compounds or adsorbable trace elements.

To quantify equilibrium sorption capacities of polar organic compounds in migrating petroleum, we have established a new method for computing RSC (*K*_*r*_ values) of polar compounds in natural migration petroleum, on the basis of Eq. (4) (Supplementary Text S-1.2). The method for computing the relative sorption coefficient involves the following steps:

The 1st step is to conduct regression analysis using Supplementary Equation (S3) instead of Eq. (1) in Zhang *et al*.^5^, to obtain estimates of the values for the constants ${a}_{1}$, ${a}_{2}$, ${a}_{3}$ and ${a}_{4}$ that are more accurate than achievable with the previous equation in Zhang *et al*.^5^. The data preparation and the subsequent non-linear regression analyses are presented in Zhang *et al*.^5^. However, the non-linear regression analyses herein are conducted in an iterative manner (Supplementary Text S-1.2) to obtain more rational regression equations.

The 2nd step is to calculate the *λ* ratios (*λ is* the relative variation rate of the concentration at the reference point for a given polar compound) from Supplementary Equation (S8), the migration-sorption factor ${a}_{\lambda }$ (a constant controlling migration-sorption fractionation) and finally the relative sorption coefficient *K*_*r*_ with Supplementary Equation (S9) and Eq. (4), respectively.

The *K*_*r*_ values of the alkylcarbazoles in the petroleum in the Xifeng Oilfield were calculated and are listed in Supplementary Table S2(Supplementary Text S-1.2).

## Supplementary information

Supplementary Information
