# Supplementary material for: Relative sorption coefficient: Key to tracing petroleum migration and other subsurface fluids
Source: Sci Rep. 2019 Nov 14;9:16845. doi: 10.1038/s41598-019-52259-6 (PMC6856530; doi:10.1038/s41598-019-52259-6)
Supplement: Supplementary file 1 — Supplementary Information [file 41598_2019_52259_MOESM1_ESM.docx]

Scientific Reports

Supplementary Information for

Relative sorption coefficient: Key to tracing petroleum migration and other subsurface fluids

Liuping Zhang, Yang Wang, Maowen Li, Qing-Zhu Yin, Wenzheng Zhang

Corresponding author: Liuping Zhang

Email: [lpzhang@mail.iggcas.ac.cn](mailto:lpzhang@mail.iggcas.ac.cn) / [lpzhang_int@sina.com](mailto:lpzhang_int@sina.com)

**This file includes:**

Supplementary text

Figs. S1 to S5

Tables S1 to S5

S-1 Supplementary Information Text

S-1.1 Sorption capacities of polar molecules in secondary petroleum migration

In natural systems of secondary petroleum migration (SPM), concentrations of polar compounds may give erroneous information about SPM if used directly, as they are also influenced by dissolution in water, organic facies and thermal maturation of source rocks, and biodegradation of petroleum^5,11-15,17-20,35-39^. When using concentrations of polar compounds to trace SPM, it is necessary to identify and to eliminate these influences (or interferences). Based on published research in the literature^1,3,5,10,13,18,41,42^, some of these influences such as dissolution in water and biodegradation of polar compounds can be avoided by selecting relatively stable polar compounds (e.g. carbazoles) with suitable sorption capacities and low water solubility, if biodegradation levels are less than 3 on the scale of Peters and Moldowan (1993)^40^. The influences from organic facies can be minimized by grouping petroleum samples according to their organic facies. The maturity effect on concentrations of the polar compounds, however, is inevitable^5,10-13,15,42^ , but can be eliminated by constructing secondary migration fractionation indices (*SMFIs*, see Zhang et al. (2013)^5^ for details).

The concentration of a given polar compound at the start point of petroleum migration or at a reference point varies with time. The extent of such variation differs between polar compounds and affects the *SMFI*s and their ratios. Therefore, the *SMFIs* need to be developed further or amended to eliminate such influence (details in the next two sections). When all the influences noted above are successfully avoided, minimized or eliminated, the amended *SMFI*s (denoted as *SMFI_λ_*s, see the following two sections) of polar compounds should decrease exponentially with increasing migration distance^5^, as should the *SMFI_λ_* ratios of the polar compounds with strong sorption capacities to those with relatively weak sorption capacities. If this is not the case, then those influences have not been eliminated correctly or completely. Therefore, *SMFI_λ_* ratios of polar compounds with distinctly different sorption capacities can help to check the validity of influence avoidance, minimization and elimination. However, these indices need to be established on the basis of sorption capacities of polar compounds in migrating petroleum.

Alkyl- and benzo-carbazoles have many advantages as petroleum migration tracers such as unambiguous structures, low solubility in water, strong polarities and suitable sorption capacities^3,6,46-48^ and thus are often employed as important tracers. But their use may become invalid for high- to post-mature or severely biodegraded petroleum, due to significant alteration by thermal cracking or biodegradation of these carbazoles^5,18,40^. In these scenarios, more stable polar compounds will need to be selected for tracing secondary migration. If sorption capacities of selected polar compounds are too high, they can only reflect short-distance migration. If their sorption capacities are too low, they cannot effectively reflect secondary migration. Therefore, the selection of more stable polar compounds also needs to consider the sorption capacities of these polar compounds in migrating petroleum.

In practice, the new understandings achieved from the study of sorption capacities of polar compounds in migrating petroleum can help guide the selection of polar compounds (that are suitable to specific areas) and the establishment of geochemical indices to trace secondary migration. They can also help to eliminate and avoid influences from source facies, maturity of organic matter and biodegradation.

**S-1.2 Calculation of the relative sorption coefficient**

The method for computing the relative sorption coefficient of polar compounds in SPM was established on the basis of the equations for migration-sorption fractionation (equations (S8, S17, S19 and S20) in Zhang et al. (2013)^5^), which were derived from the mass balance principle and the general advection-reaction-dispersion equation^30,44^. The migration-sorption fractionation equation^5^ can be written as:

$$\left\{ \begin{aligned} C(x,t)=C_{0}(t)\cdot e^{-R_{d}\cdot\lambda\cdot x/v} \\ C_{0}(t)=C_{0}(t_{0})\cdot e^{\lambda\cdot t} \end{aligned} (\text{S}1) \right.$$

where $C(x,t)$ is the concentration of a given polar compound (e.g. a carbazole) in petroleum (mg/cm^3^); $x$ is the migration distance or relative migration distance (km) from the starting point or a reference point on a secondary migration pathway (refer to Zhang et al. (2013)^5^), respectively; $t$ is the time of secondary migration (year); $C_{0}(t)$ represents the concentration of the polar compound in petroleum at the starting point of the secondary migration pathway or at a reference point (e.g. the charging point), at this point $x=0$; $t_{0}$ is the commencement time of secondary migration; $C_{0}(t_{0})$ is $C_{0}(t)$ at $t_{0}$ and is a constant (mg /cm^3^) for the polar compound in the migration pathway; $R_{d}$ represents the retardation factor of the polar compound in migrating petroleum (a dimensionless constant), being related to the migration and the equilibrium sorption of the polar compound; $v$ is the average velocity of the migration (km/year); *λ* is a constant (year^-1^) for the polar compound (discussed below).

The variation of $C_{0}(t)$ with maturity can be represented by the concentration changes of polar compounds (e.g. alkyl- and benzo-carbazoles) in source rock extracts and in petroleum close to source rocks, which vary steadily with maturity over the range of 0.45-1.2% in vitrinite reflectance equivalent (refs. 20,21). Therefore, a quadratic equation can describe most of these variations of the concentrations with maturity^5^:

$$C_{0}\left( t \right)=C_{0}(R_{o}(t))=a_{1}\cdot(1+a_{2}\cdot R_{o}(t)+a_{4}\cdot{R_{o}(t)}^{2}) (S2)$$

where $R_{o}(t)$ represents vitrinite reflectance and its equivalent ($R_{o}$(equiv.)) that quantitatively indicate the maturity levels (%). The parameter $a_{1}$ is dictated by geochemical processes of hydrocarbon generation and fractionation in primary migration or migration before the reference point, with the unit of concentration (mg/cm^3^); $a_{2}$ and $a_{4}$ determine the extent of variation of the concentration of a polar compound at the starting point or at a reference point during secondary petroleum migration (i.e., $C_{o}(t)$) with $R_{o}(t)$ and ${R_{o}(t)}^{2}$, respectively, being dimensionless.

Substitution of equation (S2) into (S1) yields:

$$C\left( t \right)=C\left( x,R_{o}\left( t \right) \right)=a_{1}\left( 1+a_{2}R_{o}\left( t \right)+a_{4}{R_{o}\left( t \right)}^{2} \right)e^{a_{3}\cdot x} \left( \text{S}3 \right)$$

$$a_{3}=-R_{d}\cdot\frac{\lambda}{v} (\text{S}4)$$

where $a_{3}$ is a constant for a polar compound (km^-1^) and is always negative when sorption occurs and there are no other chemical reactions during the secondary migration process, or the influences of the other chemical reactions are eliminated. Over a very narrow Ro (equiv.) range such as 0.7–0.8% (ΔRo~0.1%), $a_{4}$ is close to 0 and equation (S2) becomes linear. In this scenario, equation (S3) becomes equation (1) in Zhang et al. (2013)^5^.

The values of $a_{1}$, $a_{2}$, $a_{3}$ and $a_{4}$ can be determined through non-linear regression analysis of equation (S3), using the data of $C\left( x,R_{o}\left( t \right) \right)$, $R_{o}\left( t \right)$ and $x$ (refer to Zhang et al., 2013)^5^. On this basis, it seems that if $\lambda$ and $v$ are known, $K_{d}$ could be derived from equations (2 and S4). However, $K_{d}$ cannot be obtained directly from $R_{d}$ (equation (2)), because it is nearly impossible to measure or to reliably estimate the values of $\lambda$ and $v$ during petroleum migration.

The constant $\lambda$ for a given polar compound can be expressed by the following equation derived from equation (S1):

$$\lambda\cdot t=ln(C_{0}(t))-ln(C_{0}(t_{0})) (\text{S}5)$$

As $C_{0}(t_{0})$ does not vary with time, we get

$$\lambda=\frac{d(\lambda t)}{dt}=\frac{1}{C_{0}(t)}\cdot\frac{d(C_{0}(t))}{dt} (\text{S}6)$$

Therefore, $\lambda$ (year^-1^) is the relative variation rate of $C_{0}(t)$ for a given polar compound. If $\lambda$ were a constant in a migration system, $a_{3}$ would be only related to migration and sorption (i.e., $v$ and $R_{d}$) and thus would be the factor controlling the migration-sorption fractionation of a polar compound (equation (S4), refer to Zhang et al., 2013)^5^. However, $\lambda$ should be different among most polar compounds in a migration system. Therefore, although $a_{3}$ is the primary control (as shown in equations (S3-S4)) of the migration-sorption fractionation of a given polar compound, its value is influenced by $\lambda$. This influence may lead to erroneous results when migration-sorption fractionations of different polar compounds are compared.

Substitution of equation (S2) into (S6) yields

$$\lambda=\frac{a_{2}+2a_{4}R_{o}\left( t \right)}{1+a_{2}R_{o}\left( t \right)+a_{4}{R_{o}\left( t \right)}^{2}}\cdot\frac{d(R_{0}(t))}{dt} (\text{S}7)$$

From equation (S7), it is not easy to directly calculate $\lambda$ either, because it is difficult to obtain accurate $d(R_{0}(t))/dt$. For this reason, an indirect approach is established to solve the problem of calculating $\lambda$. If a compound (e.g. 3,4- or 1,8-dimethylcarbazole) is selected to serve as a reference compound, the $a_{2}$, $a_{4}$ and $\lambda$ constant of this compound are fixed in a migration system and denoted as $a_{2f}$, $a_{4f}$ and $\lambda_{f}$, respectively. Then:

$$\lambda_{f}/\lambda=\frac{{[a}_{2f}+2a_{4f}R_{o}\left( t \right)]\cdot[1+a_{2}R_{o}\left( t \right)+a_{4}{R_{o}\left( t \right)}^{2}]}{{[a}_{2}+2a_{4}R_{o}\left( t \right)]\cdot[1+a_{2f}R_{o}\left( t \right)+a_{4f}{R_{o}\left( t \right)}^{2}]} (\text{S}8)$$

where $a_{2f}$, $a_{4f}$ and $\lambda_{f}$ represent the $a_{2}$, $a_{4}$and $\lambda$ values of a reference compound (e.g. 3,4- or 1,8-dimethylcarbazole) selected for a fixed $\lambda$ in a migration system. From equation (S8), the $\lambda_{f}/\lambda$ ratio of the reference compound to any given polar compound can be easily calculated.

To eliminate the influence of $\lambda$, a new parameter termed the migration-sorption factor $a_{\lambda}$ is introduced:

$${a_{\lambda}=(\lambda}_{f}/\lambda)\cdot a_{3} (\text{S}9)$$

From equations (S4 and S9), we get:

$$a_{\lambda}=-R_{d}\cdot\frac{\lambda_{f}}{v} (\text{S}10)$$

where $a_{\lambda}$ is a constant (km^-1^), controlling migration-sorption fractionation of a polar compound. When these equations are used to study secondary migration fractionation instead of $a_{3}$, the influence of $\lambda$ is eliminated (equation (S10)) as $\lambda_{f}$ is a constant for all the studied polar compounds in a migration system. From equation (S10), we obtain:

$$R_{d}=-a_{\lambda}\cdot\frac{v}{\lambda_{f}} (\text{S}11)$$

For a series of $R_{d}$ values of polar compounds in a migration system, we have:

$$R_{dmin}=-a_{\lambda max}\cdot\frac{v}{\lambda_{f}} (\text{S}12)$$

$$R_{dmax}=-a_{\lambda min}\cdot\frac{v}{\lambda_{f}} (\text{S}13)$$

where $R_{dmax}$ and $R_{dmin}$ are the same as those in equation (3) in the Methods; $a_{\lambda max}$ is the maximum in a series of $a_{\lambda}$ values of polar compounds (km^-1^); $a_{\lambda min}$ is the minimum (km^-1^).

For a natural migration system, it is difficult to directly measure the values of $v$ in equations (S11-S13), because petroleum migration usually occurred in past geological times. The values of $v$ and $\lambda_{f}$ are the same for different compounds in a migration system, and thus are eliminated when substituting equations (S11-S13) into equation (3) in the Methods:

$$K_{r}=\frac{-a_{\lambda}\frac{v}{\lambda_{f}}+a_{\lambda max}\frac{v}{\lambda_{f}}}{-a_{\lambda min}\frac{v}{\lambda_{f}}+a_{\lambda max}\frac{v}{\lambda_{f}}}\times100\left( \% \right)$$

$$=\frac{a_{\lambda max}-a_{\lambda}}{a_{\lambda max}-a_{\lambda min}}\times100\left( \% \right) \text{(S14)}$$

Equation (S14) can be used to calculate $K_{r}$ for quantitative evaluation of equilibrium sorption capacities, even if $n$, $n_{s}$, $\rho_{s}$, $\lambda$ and $v$ are unknown. As $\lambda_{f}$ is eliminated in equation (S14), $K_{r}$ does not vary when a different reference compound is used for $\lambda_{f}$, even though the $\lambda_{f}/\lambda$ ratio varies with different reference compounds. The unique solution of the relative sorption coefficient $K_{r}$ has also been demonstrated by calculations with various reference compounds.

To obtain reliable $K_{r}$ values, rational regression equations of equation (S3) are needed. The concentrations of alkylcarbazoles at the starting point (i.e., $C_{0}(t)$) rise with the increasing $R_{o}(t)$ from 0.45% to 1.2%^13^. Because $R_{o}(t)$ does not decrease with time and $\lambda$ is not equal to 0 (ref. 5), from equation (S6)) we can deduce that the $\lambda$ constant is greater than 0 in this $R_{o}(t)$ range, which provides one of the constraining conditions for rational regression equations. From equation (S7), we can deduce that if ${[a}_{2}+2a_{4}R_{o}\left( t \right)]/[1+a_{2}R_{o}\left( t \right)+a_{4}{R_{o}\left( t \right)}^{2}]$>0, $\lambda$ must be greater than 0 because $d{(R}_{o}\left( t \right))/dt>0$. Therefore, even though the constant $\lambda$ cannot be directly obtained, we can assess whether $\lambda$>0 when a regression equation of equation (S3) is obtained. In another aspect, the $\lambda_{f}/\lambda$ ratio should not vary with $R_{o}\left( t \right)$, as $\lambda$ and $\lambda_{f}$ are constants for any given compound and the reference compound, respectively^5^. However, equation (S8) is not an accurate equation, as it is based on the regression equations of equation (S3). Therefore, the $\lambda_{f}/\lambda$ ratios calculated from equation (S8) may vary with $R_{o}\left( t \right)$. If the variation of $\lambda_{f}/\lambda$ ratios is sufficiently small, the regression equations of equation (S3) are rational and can be accepted if $\lambda$>0. The conditions of $\lambda$>0 and small variation of $\lambda_{f}/\lambda$ can be used, in turn, to examine the rationality of non-linear regression equations of equation (S3). To obtain rational non-linear regression equations, the non-linear regression analyses are conducted in an iterative manner with the conditions of $\lambda$>0 and small variation of $\lambda_{f}/\lambda$. If these conditions are not met by a non-linear regression equation, the regression analysis is performed again, until these conditions are met: $\lambda$>0，little variation of $\lambda_{f}/\lambda$, and a relatively high correlation coefficient. If these conditions cannot be met at all in the iterative calculation for a polar compound, this polar compound must be abandoned.

Here, we present an example showing the calculation of the $K_{r}$ values for alkylcarbazoles with data from the Xifeng Oilfield^5^ in the Ordos Basin, China. Fig. S1 shows the mass chromatograms of alkylcarbazoles in the petroleum samples with different relative migration distance in the Xifeng Oilfield. In this figure, the peak height of 1,8-DMCA is normalized to 100. Therefore, the peak heights or areas of the other DMCAs represent their concentrations relative to the concentration of 1,8-DMCA. According to the existing sorption theory based on the shielding and partially shielding effects, the peak heights or areas of all the DMCAs except 1,8-DMCA should decrease with increasing migration distance, such as 1,2-, 1,5-, 1,6-, 1,7-, 2,3-, 2,4-, 2,5-, 2,6- and 2,7-DMCAs. However, 1,3- and 3,4-DMCA do not show this expected trend, and instead they display an opposite trend (Fig. S1). This clearly shows that the existing theory on sorption capacities is inadequate to properly predict the behaviors of polar molecules in migrating petroleum.

In fact, concentrations of alkylcarbazoles can be affected by variations in organic facies and thermal maturation of source rocks as well as biodegradation of petroleum^10,12-15,18^. The influence from source facies and biodegradation of petroleum in the Xifeng Oilfield can be neglected^5^. The relative migration distances and thermal maturity (vitrinite reflectance equivalent) of petroleum samples from the field were calculated in Zhang et al. (2013)^5^ and are shown in Table S1.

The method for the linear regression analyses of equation (S3) is documented in Zhang et al. (2013)^5^. Here, in order to obtain more accurate results, the quadratic equation describing the relationship between $C_{0}(t)$ and $R_{0}(t)$ (equation (S2)) was used instead of the linear equation (equation (S21) in Zhang et al., 2013)^5^. The values of $a_{1}$*,*$a_{2}$,$a_{3}$ and $a_{4}$ for each alkylcarbazole (Table S2) were derived from the re-analyses of non-linear regression of alkylcarbazole concentrations given in the Table S4 in Zhang et al. (2013)^5^, the relative migration distance and $R_{0}(t)$ values in Table S1. The correlation coefficients of the regression re-analyses are significantly high, indicating strong correlations (Table S2). These regression equations satisfy the constraining condition of $\lambda$>0. Their rationality is examined further with another constraint of small variation of $\lambda_{f}/\lambda$. Table S3 shows that most coefficients of variation (standard deviation / mean×100 (%)) of the calculated $\lambda_{f}/\lambda$ ratios for each carbazole are less than 10% and the rest are smaller than 15%. All the coefficients of variation of the $\lambda_{f}/\lambda$ mean values are less than 4%. Therefore, the non-linear regression equations can be accepted, and the mean value of the $\lambda_{f}/\lambda$ ratios of each alkylcarbazole in Table S3 is used to calculate the value of the alkylcarbazole from equation (S9). The results are shown in Table S2. The relative sorption coefficients of the alkylcarbazoles were then calculated using equation (S14) from values, with the $a_{\lambda min}$ of -17.28 km^-1^ and $a_{\lambda max}$ of -0.028 km^-1^, and are also listed in Table S2. The error analysis of RSCs is presented in the section S-1.4.

**S-1.3 Modification of the secondary migration fractionation index**

To trace the distances of lateral secondary migration, the Secondary Migration Fractionation Index (*SMFI*) was defined in a previous study^5^ as:

$$SMFI=\frac{C\left( x,R_{o}\left( t \right) \right)}{C_{0}\left( R_{o}\left( t \right) \right)} \times100(\%)=e^{a_{3}x} \times100(\%) (\text{S}15)$$

Although *SMFI* can reflect the migration distance or fractionation of a polar compound and was considered an essential parameter for establishing useful ratios^5^, its value is influenced by $\lambda$, which is different from one compound to another (see the S-1.2 section of the *SI* for details). Therefore, *SMFI* cannot be used to construct ratios using different (sub)groups of polar compounds in a migration system. To eliminate the influence of $\lambda$, *SMFI* is modified as:

$$SMFI_{\lambda}=SMFI^{{(\lambda}_{f}/\lambda)/\overline{\lambda_{f}/\lambda}}=\left[ \frac{C\left( x,R_{o}\left( t \right) \right)}{C_{0}\left( R_{o}\left( t \right) \right)} \right]^{{(\lambda}_{f}/\lambda)/\overline{\lambda_{f}/\lambda}}\times100\left( \% \right) \left( \text{S16} \right)$$

where *SMFI_λ_* represents the modified *SMFI*, eliminating the influence of $\lambda$; $\overline{\lambda_{f}/\lambda}$ is the average value of the $\lambda_{f}/\lambda$ ratios of all polar compounds examined in a migration system.

Using equations (S9, S15 and S16), we can demonstrate

$$SMFI_{\lambda}=e^{{(a}_{\lambda}/\overline{\lambda_{f}/\lambda})\cdot x}\times100\left( \% \right) \left( \text{S17} \right)$$

When $SMFI_{\lambda}$ is used, the influence of $\lambda$ on *SMFI* is eliminated because $a_{\lambda}$ is not influenced by $\lambda$ (refer to equation (S10)). As noted above, $\lambda_{f}/\lambda$ may vary when a different reference compound is selected to calculate $\lambda_{f}/\lambda$. This variation does not affect the comparison of the migration fractionations between polar compounds in a migration system and the construction of indices, if the $\lambda_{f}/\lambda$ ratio is accurate. However, the $\lambda_{f}/\lambda$ ratios, derived from regression equations, have uncertainties, which may be amplified when only using $\lambda_{f}/\lambda$ (as an exponent) to eliminate the influence of $\lambda$. The extreme amplification of these uncertainties can be avoided by using ${(\lambda}_{f}/\lambda)/\overline{\lambda_{f}/\lambda}$ instead of $\lambda_{f}/\lambda$.

Equations (S15 and S17) show that both *SMFI* and *SMFI_λ_* are equal to 100% at the starting point or at a reference point (i.e. $x=0 km$). However, when equation (S2) obtained from the regression analysis of equation (S3) is directly substituted into equations (S15-S16), the *SMFI* and *SMFI_λ_* values at the starting point or a reference point (i.e. $x=0$ km) are not always equal to 100%, also due to the uncertainties of regression equations. Especially, these uncertainties may also be amplified by the power in equation (S16). Therefore, when equation (S2) obtained from the regression analysis of equation (S3) is substituted into equations (S15-S16), a correction factor $F_{C}$ for $C_{0}(t)$ needs to be added:

$$SMFI=\frac{C\left( x,R_{o}\left( t \right) \right)}{{F_{C}\cdot a}_{1}\left( 1+a_{2}R_{o}\left( t \right)+a_{4}{R_{o}\left( t \right)}^{2} \right)} \times100(\%) (\text{S}18)$$

$$SMFI_{\lambda}=\left[ \frac{C\left( x,R_{o}\left( t \right) \right)}{{F_{C}\cdot a}_{1}\left( 1+a_{2}R_{o}\left( t \right)+a_{4}{R_{o}\left( t \right)}^{2} \right)} \right]^{{(\lambda}_{f}/\lambda)/\overline{\lambda_{f}/\lambda}}\times100(\%) (\text{S}19)$$

where $F_{C}$ is a dimensionless correction factor. Let:

$$Y=\frac{C\left( x,R_{o}\left( t \right) \right)}{a_{1}\left( 1+a_{2}R_{o}\left( t \right)+a_{4}{R_{o}\left( t \right)}^{2} \right)} (\text{S20})$$

After calculating the $Y$ values, $F_{C}$ can be obtained from the regression analysis of $Y=F_{C}\cdot e^{a_{3}x}$. It can be demonstrated that adding $F_{C}$ does not affect $\lambda_{f}/\lambda$, $a_{\lambda}$ and $K_{r}$.

The other indices for tracing the distances of lateral secondary migration (equations (6-8) in Zhang et al., 2013)^5^ are also modified accordingly. For a given subgroup of alkylcarbazoles (e.g., Subgroup II-2 of dimethylcarbazoles in the main text), the *GM* index, which was previously defined as the geometric mean of *SMFI*s of alkylcarbazoles in this subgroup^5^, needs to be modified as the geometric mean of *SMFI_λ_*s:

$$GM_{\lambda}=\sqrt[n]{\prod_{i=1}^{n} {SMFI}_{\lambda i}}=e^{(\overline{a_{\lambda type}}/\overline{\lambda_{f}/\lambda})\cdot x}\times100(\%) (\text{S}21)$$

where ${GM}_{\lambda}$ is the modified *GM*, $SMFI_{\lambda i}$ is the $SMFI_{\lambda}$ of the $i$th polar compound in this subgroup (e.g., the ith alkylcarbazole in subgroup III-2), $n$ is the number of the polar compounds in the subgroup (e.g., $n$ is equal to 3 for subgroup III-2 shown in Fig. 3), and $\overline{a_{\lambda type}}$ is the arithmetic mean of the $a_{\lambda}$ values for the same type of polar compounds.

Based on equation (S21), the ${GM}_{1}/{GM}_{2}$ index (refer to equation (7) in Zhang et al. (2013)^5^) is modified as:

$${GM}_{\lambda1}/{GM}_{\lambda2}=e^{(\overline{a_{\lambda type}^{1}}-\overline{a_{\lambda type}^{2}})/\overline{\lambda_{f}/\lambda}\cdot x} (\text{S}22)$$

where ${GM}_{\lambda1}$ and ${GM}_{\lambda2}$ are the geometric means of *SMFI_λ_*s for two types of alkylcarbazoles, respectively. The types can represent subgroups here. $\overline{a_{\lambda type}^{1}}$ represents $\overline{a_{\lambda type}}$ of type one alkylcarbazoles; $\overline{a_{\lambda type}^{2}}$, type two alkylcarbazoles.

Similarly, the ratios of *SMFI_λ_*s for two different types of individual alkylcarbazoles with distinct sorption capacities can be derived from equation (S17):

$${SMFI}_{\lambda1}/{SMFI}_{\lambda2}=e^{(a_{\lambda}^{1}-a_{\lambda}^{2})/\overline{\lambda_{f}/\lambda}\cdot x} (\text{S}23)$$

where ${SMFI}_{\lambda1}$ is the ${SMFI}_{\lambda}$ of a type one alkylcarbazole; ${SMFI}_{\lambda2}$, a type two alkylcarbazole; $a_{\lambda}^{1}$ represents the $a_{\lambda}$ value of a type one alkylcarbazole; $a_{\lambda}^{2}$ the $a_{\lambda}$ value of a type two alkylcarbazole.

${SMFI}_{\lambda}$, ${SMFI}_{\lambda1}/{SMFI}_{\lambda2}$, ${GM}_{\lambda}$, and ${GM}_{\lambda1}/{GM}_{\lambda2}$ are all functions of (relative) migration distance $x$. Thus, they can all serve as odometers for secondary migration and can be used to identify migration fractionation and migration directions or pathways. Both ${SMFI}_{\lambda1}/{SMFI}_{\lambda2}$ and ${GM}_{\lambda1}/{GM}_{\lambda2}$ decrease with increasing migration distance when ${SMFI}_{\lambda1}$ and ${GM}_{\lambda1}$ are calculated from compounds of the type with stronger sorption capacities compared to those with lower sorption capacities.

In general, the molecular indices including ${SMFI}_{\lambda}$ (or $SMFI$) and ${GM}_{\lambda}$ (or *GM*) are used to reveal migration directions, routes and distances, whereas the indices ${SMFI}_{\lambda1}/{SMFI}_{\lambda2}$ and ${GM}_{\lambda1}/{GM}_{\lambda2}$ are employed to validate the migration information obtained from the molecular indices, and to check the validity of influence avoidance, minimization and elimination. In this way, the molecular indices and their ratios constitute mutual authentication in tracing secondary petroleum migration.

The alkylcarbazole data from the Xifeng Oilfield in the Ordos Basin (Tables S1, S3 and S4 in Zhang et al., 2013)^5^ are also used as an example to show the calculation of the modified *SMFI* and its related indices. The method for computing $SMFI$ values is presented in Zhang et al. (2013)^5^. On the basis of the iterative non-linear regression analysis of equation (S3) under the conditions of $\lambda$ >0 and small variation of $\lambda_{f}/\lambda$ as discussed in the section above, the factor $F_{C}$ (Table S2) was derived from equation (S20) and the related regression analyses. Most of the $F_{C}$ values are close to 1 (Table S2). $SMFI$ was calculated with equation (S18). Using the $SMFI$ values and the $\lambda_{f}/\lambda$ ratios calculated in the previous section, $SMFI_{\lambda}$ (Table S4) was computed with equation (S16). As shown in Fig. S2, the $SMFIs$ clearly display an exponential decrease with increasing relative migration distance, and the $SMFI_{\lambda}s$ also show a similar variation trend. The only difference between the two is the powers of the regression equations (Fig. S2), arising from the correction (refer to equation (S16)). The ${GM}_{\lambda}$, ${GM}_{\lambda1}/{GM}_{\lambda2}$ and $SMFI_{\lambda1}/SMFI_{\lambda2}$ values were computed using the $SMFI_{\lambda}$ values (equations (S21-S23)). The latter two ratios are established from different types of polar compounds on the basis of their sorption capacities, as discussed in the main text.

**S-1.4 Random error estimation of the relative sorption coefficient**

The RSC random error is derived from equations (S9, S14-S16). The parameters $a_{\lambda max}$ and $a_{\lambda min}$ in equation (S14) are fixed for the estimation of the RSCs of different compounds and do not introduce random error. Therefore, from equation (S14), we can get

$${\Delta K}_{r}=\frac{-\Delta a_{\lambda}}{a_{\lambda max}-a_{\lambda min}}\times100\% (\text{S}24)$$

where ${\Delta K}_{r}$ is the RSC error; and $\Delta a_{\lambda}$ is the $a_{\lambda}$ error.

If we have the values of $a_{\lambda}$ errors of polar compounds, the RSC errors can be easily calculated. For $a_{\lambda}$ errors, substitution of equation (S15) into equation (S16) yields:

$$SMFI_{\lambda}=SMFI^{{(\lambda}_{f}/\lambda)/\overline{\lambda_{f}/\lambda}}=e^{a_{3}\cdot{(\lambda}_{f}/\lambda)/\overline{\lambda_{f}/\lambda}\cdot x} (\text{S}25)$$

where $SMFI_{\lambda}$ is not multiplied by 100%.

Then, by substituting equation (S9) into equation (S25), we get:

$$SMFI_{\lambda}=e^{a_{\lambda}\cdot x/\overline{\lambda_{f}/\lambda}} (\text{S}26)$$

$$ln(SMFI_{\lambda})=a_{\lambda}\cdot x/\overline{\lambda_{f}/\lambda} (\text{S}27)$$

The average value of $\overline{\lambda_{f}/\lambda}$ does not introduce random errors, as it is a constant for all polar compounds examined in a migration system. The values of (relative) migration distance $x$ are calculated from [geodetic](javascript:;) [coordinates](javascript:;) of petroleum wells, whose errors are very small. Here, instead of the specific values of x, the average value of x is used which does not cause random errors. Then, we obtain:

$${\Delta a}_{\lambda}=\frac{\Delta ln\left( SMFI_{\lambda} \right)\cdot\overline{\lambda_{f}/\lambda}}{x} \left( \text{S}28 \right)$$

where $\Delta ln\left( SMFI_{\lambda} \right)$ is the standard deviation representing $ln\left( SMFI_{\lambda} \right)$ error.

Substituting equation (S28) into equation (S24), we get the equation for the RSC error estimation:

$${\Delta K}_{r}=\frac{-\Delta ln\left( SMFI_{\lambda} \right)\cdot\overline{\lambda_{f}/\lambda}}{(a_{\lambda max}-a_{\lambda min})\cdot x}\times100\% (\text{S29})$$

Therefore, the RSC error is proportional to $\Delta ln\left( SMFI_{\lambda} \right)$, that is, the standard deviation representing $ln\left( SMFI_{\lambda} \right)$ error.

The RSC errors of alkylcarbazoles were calculated with equation (S29) from the corresponding data listed in Tables S1-S4. The results are listed in Table S2. As , $\left\lceil{\Delta K}_{r} \right\rceil<\left| \Delta ln\left( SMFI_{\lambda} \right) \right|$. Therefore, the values of the relative sorption coefficient are more accurate, as shown in Figure 3.

**S-1.5 Shielding effect**

In Table S2, 4-methylcarbazole (MCA) has a higher relative sorption coefficient than 1- MCA. The mean value of the relative sorption coefficients of DMCAs (dimethylcarbazoles) decreases in the following order: N-H exposed DMCAs (Group III) > N-H partially shielded (or N-H partially exposed) DMCAs (Group II) > N-H shielded DMCA (i.e. 1,8-DMCA) (Group I). These calculated relative sorption coefficients ($K_{r}$ values) appear to support the previously proposed theory based on the shielding and partial shielding effects. However, when examining Table S2 in detail, we find that the $K_{r}$ values of some N-H partially shielded DMCAs (Group II) are actually close to or greater than those of most N-H exposed DMCAs (Group III). Even the $K_{r}$ value of 3,4-DMCA in Group III is unexpectedly lower than that of 1,8-DMCA (Group I). Therefore, the shielding and partial shielding effects cannot fully explain the sorption capacities of polar compounds.

Upon close examination of the ratios of polar compounds, we also find that some ratios constructed on the basis of shielding and partial shielding effects do not effectively reflect secondary migration distance. For example, the ratio of *SMFI_λ_* of 1,4-DMCA in Group II to *SMFI_λ_* of 1,8-DMCA in Group I does not show a clear decreasing trend with increasing migration distance (Fig. 2A). The ratios of *SMFI_λ_* of 2,4-DMCA in Group III to *SMFI_λ_* of 1,6-DMCA in Group II, 3,4-DMCA in Group III to 1,8-DMCA in Group I, 3,4-DMCA in Group III to 1,7-DMCA in Group II, 2,5-DMCA in Group III to 1,2-DMCA in Group II, 2,4-DMCA in Group III to 1,2-DMCA in Group II and 2,7-DMCA in Group III to 1,2-DMCA in Group II increase unexpectedly with increasing migration distance (Figs. 2D-I). The strong correlations indicate that such phenomena, which are inconsistent with predictions from the previously-proposed theory about sorption capacities of polar compounds, do not arise from analytical uncertainties.

It is clear from the above discussion that the shielding and partial shielding effects are not the only factors controlling equilibrium sorption capacities of polar compounds in migrating petroleum. Therefore, the existing theory on sorption capacities of polar molecules needs to be revised.

**S-1.6 Stripping effect**

Here, we compare desorption of polar compounds adsorbed on mineral surfaces under static conditions with that under the conditions of lateral migration. In the static scenario, bulk petroleum and the diffuse layer are all motionless at a macroscopic scale (Fig. S3A) and the sorption and desorption of a molecule occur through molecular exchange in the diffuse layer^45^. When petroleum is flowing, the diffuse layer moves and becomes much thinner (Fig. S3B), and sorption and desorption become much faster (refer to Li et al., 2011^)45^. The movement of the diffuse layer causes the molecules adsorbed on mineral surfaces to desorb much more easily than under static conditions. This stripping effect increases desorption rate relative to sorption rate and thus reduces equilibrium sorption capacities of polar molecules during petroleum migration. “Tall” polar compounds suffer from stronger stripping effect than the “shorter” ones. Therefore, the stripping effect greatly reduces equilibrium sorption capacities of alkylcarbazoles with the alkyls at positions 4 and 5 (Figs. S4-S5), which are reflected by the $K_{r}$ values.

The alkylcarbazoles in Group II are all affected by the partial shielding effect that makes the adsorbed alkylcarbazoles tilt in the absence of the alkyl at position 8 (Fig. 1 and Fig. S4). This tilting of molecules increases the height of the methyl at position 4 but decreases that at position 5. Therefore, 1,4-dimethylcarbazole is subject to a stronger stripping effect than 1,5-dimethylcarbazole, so that the sorption capacity of 1,4-dimethylcarbazole is weaker than that of 1,5-dimethylcarbazole, as shown by the relative sorption coefficient in Figs. S4A-B. The molecular tilting arising from the alkyl at position 1 also increases the height of the methyl at position 3, leading to an enhanced stripping effect on 1,3-dimethylcarbazole that significantly reduces its equilibrium sorption capacity (Fig. S4C). The other dimethylcarbazoles in Group II are all shorter than 1,4-, 1,5- and 1,3-dimethylcarbazoles and are less affected by stripping. As discussed in the main text, the dimethylcarbazoles in Group II can be divided into three subgroups, based mainly on the stripping effect.

For Group III, the stripping effect can also make molecules tilt before desorption. When 3,4-dimethylcarbazole tilts, the height of the methyl at position 3 can be increased, enhancing the stripping effect on the molecule and causing it to desorb more readily (Fig. S5A). This two-methyl stripping effect is much stronger than the one-methyl stripping effect suffered by 2,4- and 2,5-DMCAs (Figs. S5A-C). As a result, 3,4-dimethylcarbazole has the lowest $K_{r}$ value of all the dimethylcarbazoles examined here, including 1,8-dimethylcarbazole whose sorption capacity was considered to be the weakest in all the DMCAs according to the shielding effect.

**S-1.7 Impeding effect**

The Van der Waals radius of a methyl is about 0.20 nm (ref. 10) (Fig. 1). The methyls at positions 2 and 7 can impede the movement and rotation of the DMCA when it is close to the mineral surface (Fig. 1 and Fig. S5D). As a result, it becomes difficult for the hydrogen atom of the pyrrolic N-H to align with active sites on the surface for hydrogen bond formation (i.e., sorption). The methyls at positions 2 and 7 may also provide a repulsive force that weakens the hydrogen bond. These influences related to the methyls at positions 2 and 7 are hereby collectively referred to as the impeding effect.

The impeding effect can also significantly reduce equilibrium sorption capacities. The relative sorption coefficient of 2,7-DMCA is much lower than those of 2,3- and 2,6-DMCA (Fig. 3 and Figs. S5D-F), and even lower than that of 1,2-DMCA in Group II (Figs. S5D and S4F). Because of the significant reduction in sorption capacity arising from the impeding effect, 2,7-DMCA was put into Subgroup III-2 as discussed in the main text, although it is a pyrrolic N-H exposed DMCA without the methyls at positions 4 and 5.

The partial impeding effect related to the methyl at position 2 or 7 can also reduce the equilibrium sorption capacities of DMCAs within subgroups. In Subgroup II-2, 1,7-DMCA is affected by the partial shielding and partial impeding effects and has a lower relative sorption coefficient than 1,2-DMCA. The latter is mainly affected by the partial shielding effect, because the methyl at position 1 obstructs the partial impeding effect of the methyl at position 2 (Figs. S4E-F).

The partial impeding effect of the methyl at position 2 can also make the adsorbed alkylcarbazoles slightly tilt in the absence of the alkyl at position 7 (Fig. 1 and Fig. S5B). As a result, the relative sorption coefficient of 2,4-DMCA in Subgroup III-2 is significantly lower than that of 2,5-DMCA, although they both suffer from one-methyl stripping and partial impeding effects (Figs. S5B and C).

**S-1.8 Previously-proposed ratios**

As discussed above and also in the main text, many ratios based on the previously-proposed theory about sorption capacities cannot effectively explain the direction, route and distance of secondary petroleum migration (Fig. S3). To reveal the reasons, we analyze the relationships between the relative sorption coefficients of DMCAs and molecular structures.

Many DMCAs are subject to more than one effect in natural migration systems. Due to the combined effect of one-methyl stripping and partial shielding, the equilibrium sorption capacities of 1,3-, 1,4- and 1,5-DMCAs in Group II (Figs. S4A-C) are greatly reduced. In particular, the equilibrium sorption capacity of 1,4-DMCA becomes close to that of 1,8-DMCA in Group I due to the shielding effect (Fig. 3, refer to Fig. 1). As a result, the ratio of the *SMFI_λ_* of 1,4-DMCA to that of 1,8-DMCA does not show a significant decreasing trend with increasing migration distance (Fig. 2A), even though the numerator is a Group II DMCA and the denominator is a Group I DMCA.

2,4-DMCA suffers from the one-methyl stripping and partial impeding effects, whereas 1,5-DMCA is affected by the one-methyl stripping and partial shielding effects. Due to the tilting of 1,5-DMCA caused by the partial shielding effect, the stripping effect on 1,5-DMCA is reduced and becomes weaker than the striping effect on 2,4-DMCA (Figs. S5B and S4B). However, the partial shielding effect on 1,5-DMCA is stronger than the partial impeding effect on 2,4-DMCA, which can be illustrated by the fact that the shielding effect (on 1,8-DMCA) is stronger than the impeding effect (on 2,7-DMCA) (Fig. 3). Therefore, the relative sorption coefficients of 2,4-DMCA and 1,5-DMCAs are close to each other, and the ratio of their *SMFI_λ_*s does not decrease significantly with increasing migration distance (Fig. 2B). The equilibrium sorption capacity of 1,6-DMCA is higher than that of 1,5-DMCA, due to the much stronger stripping effect on 1,5-DMCA than on 1,6-DMCA. The ratio of the *SMFI_λ_* of 2,4-DMCA to that of 1,6-DMCA increases significantly with increasing migration distance (Fig. 2D), from which wrong information about migration direction would be obtained if only the partial shielding effect was considered. Clearly, it is the complex interplay of various effects that ultimately determines the equilibrium sorption capacities of these polar compounds, which explains why many previously proposed ratios failed as tracers for secondary petroleum migration (Fig. 2).

Although the ratio of *SMFI_λ_* of 2,4-DMCA to that of 1,6-DMCA significantly rises with increasing migration distance (Fig. 2D), the ratio of *SMFI_λ_* of 2,5-DMCA to that of 1,6-DMCA displays a slightly decreasing trend with increasing migration distance (Fig. 2C). This can be explained by the tilting of the molecule arising from the partial impeding effect, which weakens the stripping effect on 2,5-DMCA and causes the sorption capacity of 2,5-DMCA to become higher than that of 2,4-DMCA.

As discussed in section S-1.5, the stripping effect can be enhanced by two methyls at and near the methylation positions opposite to the N-H. The relative sorption coefficients suggest that the stripping effect on 3,4-DMCA is much stronger than the shielding effect on 1,8-DMCA. Therefore, the ratio of *SMFI_λ_* of 3,4-DMCA to that of 1,8-DMCA rises with the increasing migration distance (Fig. 2E). The shielding effect on 1,8-DMCA, however, is stronger than the combined effect of the partial shielding and partial impeding effects on 1,7-DMCA, because the partial shielding effect is stronger than the partial impeding effect. As a result, 1,7-DMCA has a higher equilibrium sorption capacity than 1,8-DMCA and 3,4-DMCA. The ratio of the *SMFI_λ_* of 3,4-DMCA to that of 1,7-DMCA displays a very clear increasing trend with migration distance (Fig. 2F).

In terms of reducing equilibrium sorption capacities of polar molecules, the partial shielding effect must be similar to the one-methyl stripping effect, because the equilibrium sorption capacity of 1,4-DMCA, which is affected by both the stripping and partial shielding effects, is close to that of 1,8-DMCA which is only affected by the shielding effect. The combined effect of the stripping and partial impeding on 2,4- and 2,5-DMCA should be stronger than the partial shielding effect on 1,2-DMCA. Therefore, the sorption capacities of 2,4- and 2,5-DMCA are weaker than that of 1,2-DMCA (Fig. 3, Figs. S5B-C and S4F). As a result, the ratios of the *SMFI_λ_*s of 2,4- and 2,5- DMCA in Group III to the *SMFI_λ_* of 1,2- DMCA in Group II increase with migration distance (Figs. 2G-H). As noted in the previous section, the sorption capacity of 2,7-DMCA in Subgroup III-2 is weaker than that of 1,2-DMCA in Group II (Figs. S5D and S4F). The ratio of the *SMFI_λ_* of 2,7- DMCA in Group III to that of 1,2- DMCA in Group II also rises with increasing migration distance (Fig. 2I). Therefore, wrong information about secondary migration would be obtained from these indices if only the partial shielding effect was considered.

Because the shielding and partial shielding effects are not the only factors that control the equilibrium sorption capacities of alkylcarbazoles in migrating petroleum, many relationships between previously established indices and migration distances appear unreasonable (Fig. 2). However, as we have demonstrated above, these seemingly unreasonable phenomena are in fact reasonable and can be explained by considering not only the previously recognized effect (i.e., shielding) but also the newly discovered effects (i.e., stripping and impeding) and the relative sorption coefficient. Therefore, many of the previously proposed ratios derived from the consideration of the shielding and partial shielding alone cannot provide reliable information about secondary petroleum migration and their use should be discontinued.

**S-1.9 New ratio indices**

In light of the new finding that the stripping, shielding and impeding effects collectively control the equilibrium sorption capacities of alkylcarbazoles in migrating petroleum, we regrouped alkylcarbazoles and established a new sequence of them according to their equilibrium sorption capacities (see the main text). Based on this new sequence of alkylcarbazoles, we established eighteen new ratios using alkylcarbazoles from different subgroups and Group I (except those between subgroups III-2 and II-3 and between II-1 and I) with distinctly different sorption capacities, so as to ensure that the ratios vary significantly with migration distance. Because the sorption capacities of the numerators of these new ratios are much stronger than those of the denominators, these ratios should decrease exponentially with increasing migration distance, and therefore can be used to verify migration fractionations inferred from the *SMFI*s or *SMFI_λ_*s, and to evaluate the influence elimination, avoidance and minimization. The corresponding ratios of geometric means of *SMFI_λ_*s among these subgroups and Group I can also serve as the parameters or odometers for secondary petroleum migration. When the sorption capacities of the numerators are significantly weaker than those of the denominators, their ratios will increase exponentially with increasing migration distance.

As shown in Fig. 4, all the newly-constructed ratios decrease exponentially with migration distance. It is worth noting that the ratios established using different DMCAs within Group II (i.e. II-3 to II-2, II-3 to II-1 and II-2 to II-1, shown in Figs. 4G, H, K and L) and within Group III (i.e. III-3 to III-2, shown in Fig. 4D) also show a clear decreasing trend with increasing migration distance. These ratio variations clearly demonstrate that effective indices for secondary migration distance can equally well be constructed from alkylcarbazoles within Group II and within Group III. Differences in equilibrium sorption capacities within each group are controlled by the stripping and/or impeding effect, but not by the shielding effect.

Besides alkylcarbazoles, the stripping effect can also be used to explain the equilibrium sorption capacities of benzocarbazoles in migrating petroleum. Because benzo[c]carbazole adsorbed on a mineral surface extends to a greater molecular height than adsorbed benzo[a]carbazole (Fig. 1 in Larter et al., 1996)^1^, benzo[c]carbazole suffers from a greater stripping effect compared with benzo[a]carbazole. As both benzo[a]carbazole and benzo[c]carbazole do not have alkyls, the difference in their equilibrium sorption capacities is mainly due to this stripping effect. The equilibrium sorption capacity of benzo[a]carbazole must be higher than that of benzo[c]carbazole, which is supported by the relative sorption coefficients of benzocarbazoles (Table S5).

**S-1.10 Significance of the relative sorption coefficient for other studies**

As discussed in section S-1.1, carbazoles may become useless if degraded or decomposed. For example, when biodegradation levels are equal to or above 3 on the scale of Peters and Moldowan^40^, alkylcarbazoles can be microbially altered and cannot serve as tracers^18^. Therefore, more stable and effective polar compounds are needed in such cases in order to trace petroleum secondary migration. To select more stable and effective polar compounds, other polar molecules, such as alkyl-dibenzothiophenes, alkyl-dibenzofurans, hydroxy-benzocarbazoles and benzothiophene-carbazoles, need to be systematically evaluated to establish a sequence of polar compounds according to their equilibrium sorption capacities. The relative sorption coefficient as defined in equation (1) and derived from equation (3 or S14) can play a pivotal role in establishing this sequence. Polar compounds that are found to be more stable and effective than alkylcarbazoles on the basis of this sequence can serve as more robust tracers for secondary petroleum migration and will be more widely applicable in various basins around the world. Furthermore, based on this sequence, the relationship between equilibrium sorption capacities and molecular structures can be analyzed to develop an understanding of the controls on the equilibrium sorption capacities of these polar compounds. This will allow proper ratios to be constructed to validate the newly-selected molecular indices and to verify secondary migration directions, routes and distances inferred from the indices of individual molecules. Therefore, the relative sorption coefficient offers an important tool to help select proper polar compounds, to understand their equilibrium sorption capacities, and to construct new ratios that can be applied to study petroleum secondary migration in basins around the world.

Sorption of polar organic compounds onto solid surfaces is of great interest to researchers in diverse fields ranging from petroleum production, transportation, and processing to biology^23,53^. The concept of the relative sorption coefficient and its computation method presented in this paper are established based on observations of natural systems rather than artificially simulated processes in lab experiments and thus more directly reflect natural processes. This approach may be useful in the fields mentioned above. For example, the concept of RSC and its computation method may be applied to investigate the adsorption of asphaltenes in petroleum production.

The concept of RSC is established herein on the basis of the linear isotherm sorption model that is widely used in many disciplines, as long as the application conditions of the model that are noted in the main text are met. The RSC evaluation method presented in this paper is derived from the migration-sorption fractionation equation (equation (S1)) for secondary petroleum migration. This migration-sorption fractionation equation was established from the linear isotherm sorption model, the mass balance principle, and the general advection-reaction-dispersion equation^5,30,31^. All these are also the foundation for studying groundwater migration and the movement of contaminants in groundwater^30,44^. Thus, the relative sorption coefficient and its evaluation method in this study should be helpful for quantitative evaluation of equilibrium sorption capacities of pollutants or tracers in natural groundwater in water resources and pollution studies.

More broadly, the migration of geofluids (such as groundwater, geothermal fluids, basinal brines, petroleum, natural gas or magmatic fluids) plays an important role in many geological processes besides the formation of petroleum pools. These geological processes generally include (1) solute transport (e.g. metal elements and organic compounds), (2) formation of ore deposits, (3) heat transport, and (4) mass wasting and tectonic movements^1,5,8,49-51^. Since the 1970s, research on geofluids has grown into a broadly acknowledged field with fundamental importance for virtually all the subdisciplines of geosciences^52^. For economic resources of noble metals used in everyday life, the corresponding elements are also enriched by geofluid migration through carrier beds from their sources in past geological times^49,50^. Tracing geofluid migration, based on sorption of geotracers such as rare earth elements in migrating geofluids, may greatly facilitate the research in the related subdisciplines of geosciences. The relative sorption coefficient and its evaluation method developed in this study should also be helpful for quantitative assessment of the equilibrium sorption capacities of adsorbable trace elements in other geofluids, which could lay a key foundation for tracing the migration of the related geofluids.

S-2 Supporting Information Figures

Fig. S1. Mass chromatograms of alkylcarbazoles (m/z=194.50-195.50) in the petroleum samples collected from two different locations in the Xifeng Oilfield, representing relative migration distances of 51.48 km (A) and 89.85 km (B), respectively. DMCA: dimethylcarbazole; ETCA: ethylcarbazole; TMCA: trimethylcarbazole.


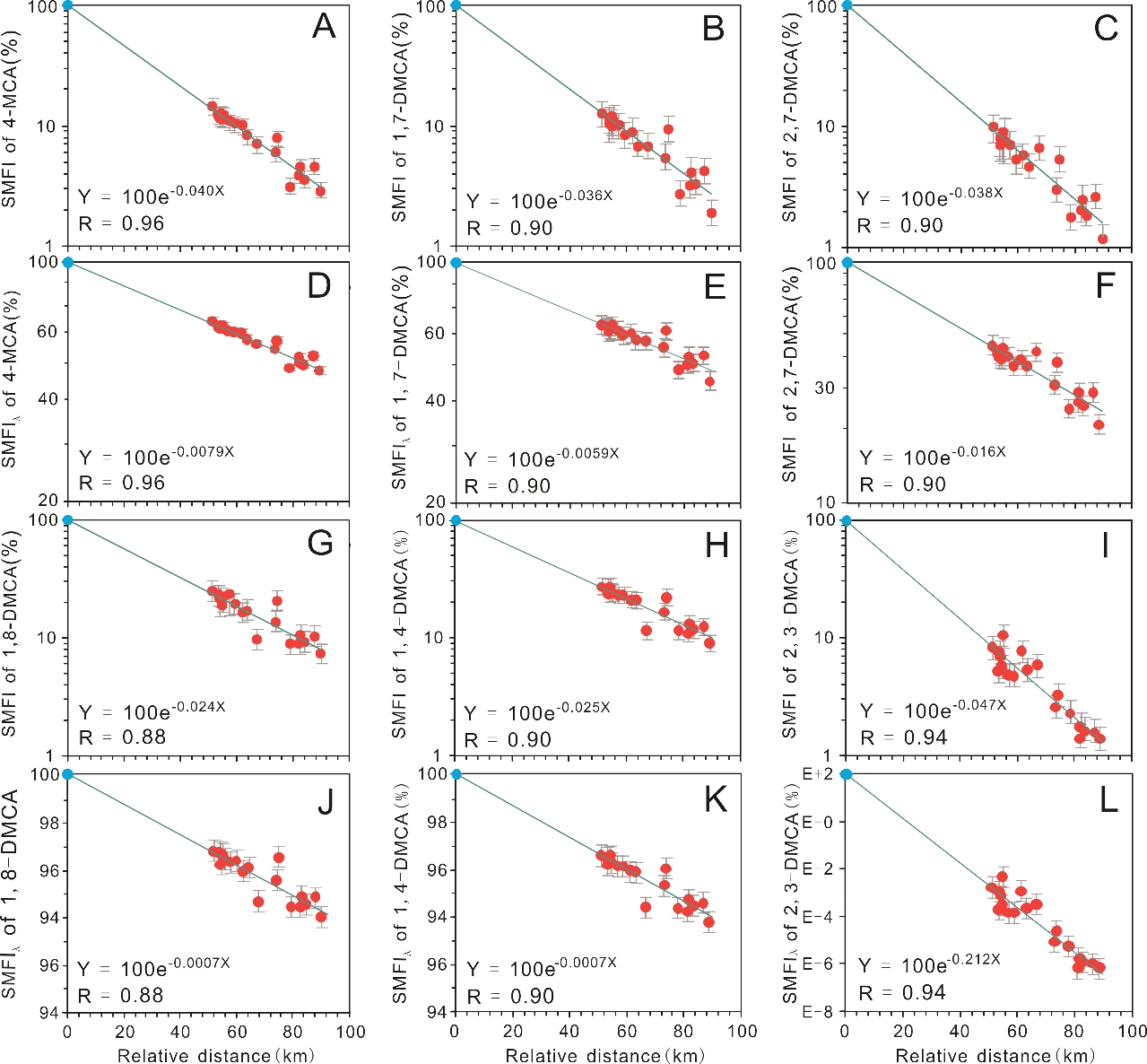


Fig. S2. Correlation diagrams showing the relationships of *SMFI* and *SMFI_λ_* values of alkyl carbazoles with relative migration distances in the Xifeng Oilfield. R: correlation coefficient; *SMFI:* secondary migration fractionation index, calculated from equation (S18) and quadratic regression equations of equation (S3) instead of the linear regression equations in Zhang et al. (2013)^5^; *SMFI_λ_*: amended *SMFI* with equation (S19); MCA: methylcarbazole; DMCA: dimethylcarbazole. The *SMFI* and *SMFI_λ_* values of 100% at the reference point ($\boldsymbol{x=0}$ km) are the model values, which were excluded in the regression analyses shown in this figure. The number of data points in each panel is nineteen; the grey error bars indicate one standard deviations (1σ) from the logarithmic values of *SMFI*s and *SMFI_λ_*s.

(A) (B)

Fig. S3. A conceptual diagram of molecule exchange for adsorption and desorption under the conditions of both static (A) and flowing petroleum (B) (after Li et al., 2011)^42^.


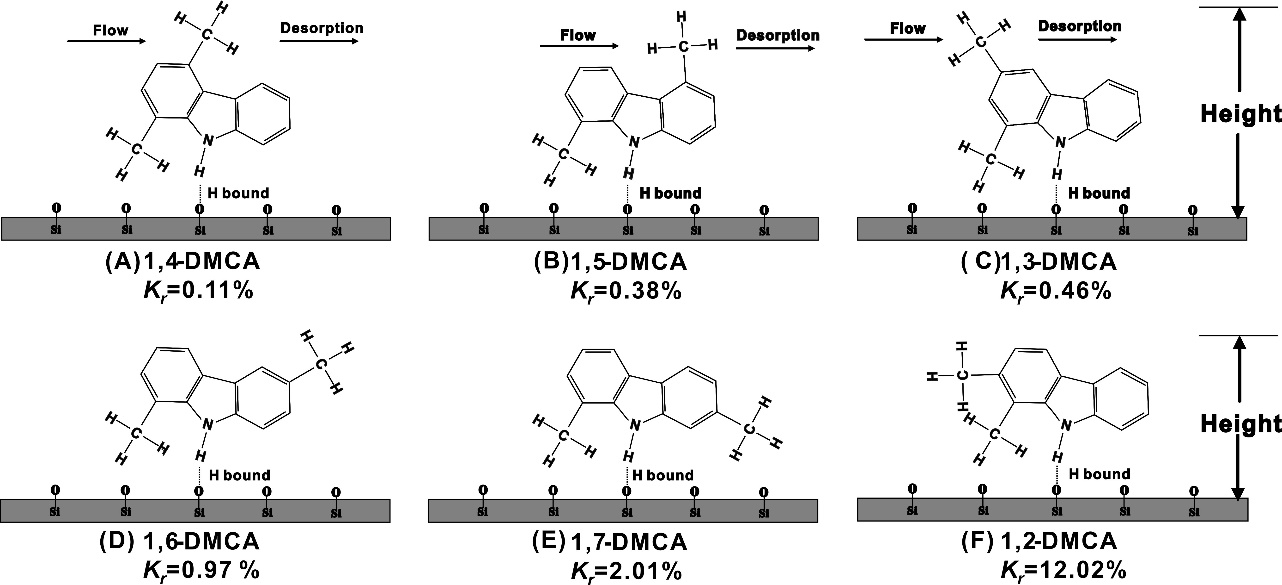


Fig. S4. H bond between electronegative atoms (e.g., O) on the mineral surface and H of N-H partially shielded dimethylcarbazoles (Group II), and desorption under the influence of petroleum flow. DMCA = dimethylcarbazole; *K_r_* = relative sorption coefficient. 1,3-, 1,4- and 1,5-dimethylcarbazoles with greater molecular heights can be more easily removed by petroleum flow than the others in Group II.


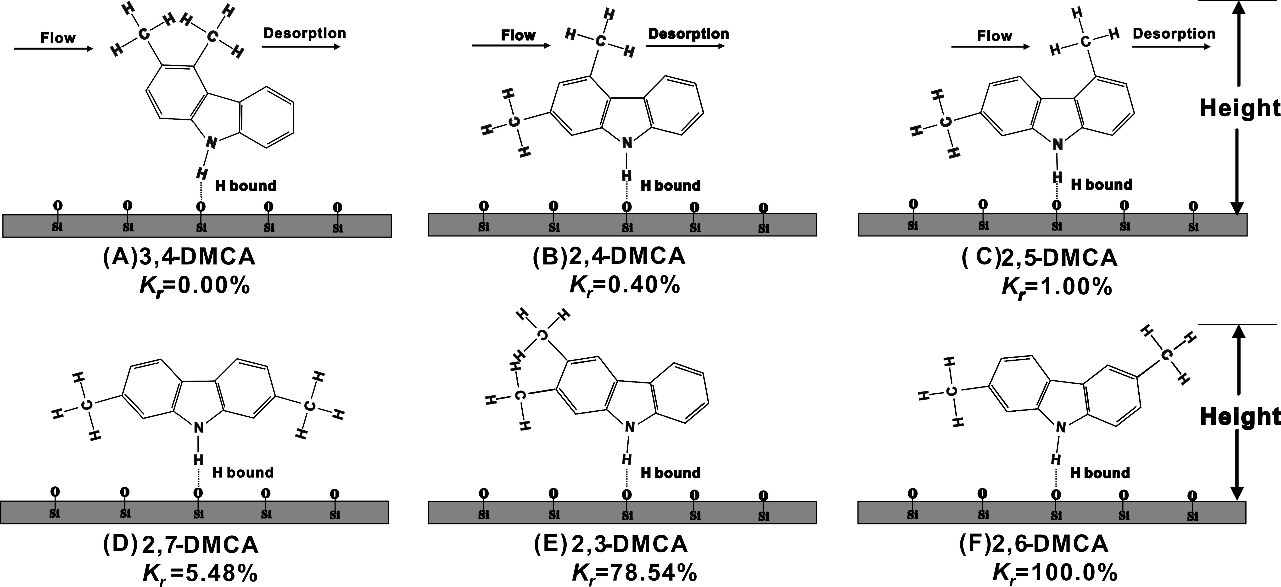


Fig. S5. H bond between electronegative atoms (e.g. O) on the mineral surface and H of N-H exposed dimethylcarbazoles (Group III), and desorption under the influence of petroleum flow. DMCA = dimethylcarbazole; *K_r_* = relative sorption coefficient. The DMCAs with the methyls at the positions opposite to N-H extend to greater molecular heights from the surface and are more easily stripped by petroleum flow than the others in this group.

S-3 Supporting Information Tables

Table S1. Saturate/aromatic hydrocarbon ratios, summary parameters from saturate GC data and Ro (equiv.) for the studied petroleum samples from the Xifeng Oilfield*.

| Sample No. | Well | Depth (m) | RD (km) |  | C_range_ | C_max_ | Ro  (equiv.) |
| --- | --- | --- | --- | --- | --- | --- | --- |
| 1 | X130 | 1303.36 | 89.85 | 1.71 | 11-34 | 20 | 0.688 |
| 2 | D68-54 | 1268.02 | 87.48 | 2.29 | 11-31 | 20 | 0.696 |
| 3 | D58-70 | 1325.93 | 84.02 | 1.58 | 11-32 | 20 | 0.690 |
| 4 | X44-039 | 1278.10 | 82.59 | 2.31 | 11-31 | 19 | 0.687 |
| 5 | X161 | 1352.32 | 82.19 | 2.64 | 11-32 | 20 | 0.696 |
| 6 | X110 | 1365.11 | 78.76 | 1.93 | 11-32 | 20 | 0.697 |
| 7 | X33-26 | 1387.71 | 74.54 | 1.41 | 11-34 | 19 | 0.703 |
| 8 | X34-023 | 1397.27 | 73.82 | 2.30 | 11-31 | 19 | 0.693 |
| 9 | X28-09 | 1409.62 | 67.28 | 1.82 | 11-33 | 20 | 0.701 |
| 10 | X167 | 1395.71 | 63.79 | 2.65 | 11-31 | 19 | 0.693 |
| 11 | X28-8 | 1392.21 | 61.93 | 2.31 | 11-32 | 20 | 0.710 |
| 12 | X27-17 | 1397.69 | 59.28 | 1.63 | 11-31 | 20 | 0.728 |
| 13 | X27-23 | 1401.56 | 57.26 | 1.22 | 11-33 | 20 | 0.734 |
| 14 | X26-28 | 1406.91 | 55.31 | 1.27 | 11-32 | 18 | 0.755 |
| 15 | X21-25 | 1433.08 | 54.97 | 2.60 | 11-31 | 20 | 0.759 |
| 16 | X30-34 | 1289.22 | 54.58 | 1.82 | 11-31 | 19 | 0.750 |
| 17 | X33-39 | 1407.41 | 53.92 | 1.55 | 11-32 | 19 | 0.751 |
| 18 | X27-35 | 1427.83 | 53.59 | 1.96 | 11-31 | 18 | 0.757 |
| 19 | X29-43 | 1437.54 | 51.48 | 2.51 | 11-32 | 20 | 0.771 |

*GC: gas chromatography; Depths are in meters below sea level; RD: Relative migration distance; Sat/Arom: ratio of saturate/aromatic hydrocarbons; C_range_: distribution range of *n*-alkanes; C_max_: the *n*-alkane with the maximum peak area; *Ro* (equiv.) represents vitrinite reflectance equivalent (%), which was derived from: 0.14(4,6-DMDBT/1,4-DMDBT)+0.57as calculated in Zhang et al. (2013)^5^.

Table S2. Calculation results of non-linear regression analyses, correction factor, migration-sorption factor and relative sorption coefficient of alkylcarbazoles in the studied petroleum samples in the Xifeng Oilfield*.

| Alkyl-  carbazoles | $lnC=ln[a_{1}(1+a_{2}Ro+a_{4}{Ro}^{2})]+a_{3}x$ | | | | | $F_{c}$ | $a_{\lambda}$ | $K_{r}$ |
| --- | --- | --- | --- | --- | --- | --- | --- | --- |
|  | $a_{1}$ | $a_{2}$ | $a_{4}$ | $a_{3}$ | r |  |  |  |
| 1-MCA | 20.90 | 0.350 | 0.190 | -0.033 | 0.909 | 1.01 | -0.391 | 2.10±0.03 |
| 4-MCA | 4.063 | 9.372 | -5.086 | -0.040 | 0.955 | 1.02 | -0.562 | 3.10±0.08 |
| 1,8-DMCA | 3.358 | -3.185 | 24.13 | -0.024 | 0.930 | 1.04 | -0.046 | 0.10±0.01 |
| PEDMCA Mean | - | - | - | - | - | - | -0.487 | 2.66±0.13 |
| EDMCA Mean | - | - | - | - | - | - | -5.360 | 30.90±1.65 |
| 1,4-DMCA | 3.503 | -3.034 | 18.21 | -0.025 | 0.953 | 1.02 | -0.048 | 0.11±0.01 |
| 1,5-DMCA | 4.997 | 6.857 | 6.478 | -0.030 | 0.950 | 1.09 | -0.093 | 0.38±0.02 |
| 1,3-DMCA | 4.663 | 7.247 | 1.525 | -0.027 | 0.934 | 1.10 | -0.108 | 0.46±0.03 |
| 1,6-DMCA | 12.36 | 2.091 | -0.053 | -0.029 | 0.927 | 1.19 | -0.196 | 0.97±0.05 |
| 1,7-DMCA | 9.858 | 6.784 | -3.348 | -0.032 | 0.923 | 1.29 | -0.375 | 2.01±0.10 |
| 1,2-DMCA | 10.48 | 0.020 | 0.050 | -0.034 | 0.905 | 1.41 | -2.101 | 12.02±0.58 |
| 3,4-DMCA | 0.028 | -368.6 | 773.3 | -0.028 | 0.813 | 1.10 | -0.028 | 0.00±0.02 |
| 2,4-DMCA | 5.158 | 0.316 | 3.197 | -0.030 | 0.925 | 1.14 | -0.098 | 0.40±0.03 |
| 2,5-DMCA | 9.313 | 1.044 | 0.484 | -0.032 | 0.918 | 1.22 | -0.201 | 1.00±0.05 |
| 2,7-DMCA | 19.60 | 0.064 | 0.095 | -0.032 | 0.895 | 1.39 | -0.974 | 5.48±0.27 |
| 2,3-DMCA | 6.446 | 0.010 | 0.005 | -0.042 | 0.869 | 1.00 | -13.578 | 78.54±2.75 |
| 2,6-DMCA | 10.67 | 0.010 | 0.001 | -0.036 | 0.822 | 1.86 | -17.281 | 100.00±6.80 |

* See the S-1.3 and S-1.4 sections of the *SI Appendix* for detailed calculation. MCA: methylcarbazole;, DMCA: dimethylcarbazole; EDMCA: N-H exposed DMCA; PEDMCA: N-H partially exposed DMCA or N-H partially shielded DMCA. The unit of $a_{1}$is μg/g; $a_{3}$ and $a_{\lambda}$, km^-1^, $\mathrm{where}a_{\lambda}=a_{3}\cdot\lambda_{f}/\lambda$ (Eq. (4) in Methods); $a_{2}$ and $a_{4}$ are dimensionless; r represents the correlation coefficient; $F_{c}$ is the correction factor for ${SMFI}_{\lambda}$ computation; $K_{r}$ is the relative sorption coefficient (%). The errors of the relative sorption coefficient were calculated with equation (S29) in *SI Appendix*, SI Text S1-1.4.

Table S3. Calculation results of the $\boldsymbol{\lambda}_{\boldsymbol{f}}\boldsymbol{/\lambda}$ ratios of each alkylcarbazole with the $\boldsymbol{R}_{\boldsymbol{0}}\boldsymbol{(t)}$ values of the studied petroleum in the Xifeng Oilfield*.

| No. | Ro  (equiv.) | 1-  MCA | 4-  MCA | 1,8-  DMCA | 1,4-  DMCA | 1,5-  DMCA | 1,3-  DMCA | 1,6-  DMCA | 1,7-  DMCA | 1,2-  DMCA | 3,4-  DMCA | 2,4-  DMCA | 2,5-  DMCA | 2,7-  DMCA | 2,3-  DMCA | 2,6-  DMCA |
| --- | --- | --- | --- | --- | --- | --- | --- | --- | --- | --- | --- | --- | --- | --- | --- | --- |
| 1 | 0.69 | 13.3 | 13.0 | 2.09 | 2.10 | 3.41 | 4.40 | 7.33 | 11.5 | 71.6 | 1.00 | 3.55 | 6.98 | 34.4 | 366.5 | 542.8 |
| 2 | 0.70 | 12.9 | 13.1 | 2.05 | 2.05 | 3.34 | 4.30 | 7.16 | 11.5 | 68.9 | 1.00 | 3.45 | 6.79 | 33.1 | 353.8 | 525.7 |
| 3 | 0.69 | 13.2 | 13.0 | 2.08 | 2.09 | 3.39 | 4.37 | 7.29 | 11.5 | 70.9 | 1.00 | 3.52 | 6.93 | 34.0 | 363.2 | 538.4 |
| 4 | 0.69 | 13.4 | 13.0 | 2.09 | 2.10 | 3.42 | 4.41 | 7.35 | 11.5 | 72.0 | 1.00 | 3.56 | 7.01 | 34.5 | 368.1 | 545.0 |
| 5 | 0.70 | 12.9 | 13.1 | 2.05 | 2.05 | 3.34 | 4.30 | 7.16 | 11.5 | 68.9 | 1.00 | 3.45 | 6.79 | 33.1 | 353.8 | 525.7 |
| 6 | 0.70 | 12.9 | 13.1 | 2.04 | 2.05 | 3.33 | 4.29 | 7.14 | 11.5 | 68.6 | 1.00 | 3.44 | 6.76 | 33.0 | 352.3 | 523.7 |
| 7 | 0.70 | 12.6 | 13.2 | 2.01 | 2.02 | 3.27 | 4.22 | 7.02 | 11.5 | 66.6 | 1.00 | 3.37 | 6.63 | 32.1 | 343.4 | 511.7 |
| 8 | 0.69 | 13.1 | 13.1 | 2.06 | 2.07 | 3.37 | 4.34 | 7.22 | 11.5 | 69.9 | 1.00 | 3.49 | 6.86 | 33.6 | 358.5 | 532.0 |
| 9 | 0.70 | 12.7 | 13.2 | 2.02 | 2.03 | 3.29 | 4.25 | 7.06 | 11.5 | 67.3 | 1.00 | 3.40 | 6.67 | 32.4 | 346.3 | 515.6 |
| 10 | 0.69 | 13.1 | 13.1 | 2.06 | 2.07 | 3.37 | 4.34 | 7.22 | 11.5 | 69.9 | 1.00 | 3.49 | 6.86 | 33.6 | 358.5 | 532.0 |
| 11 | 0.71 | 12.3 | 13.4 | 1.98 | 1.99 | 3.21 | 4.15 | 6.89 | 11.5 | 64.5 | 1.00 | 3.30 | 6.48 | 31.1 | 333.5 | 498.4 |
| 12 | 0.73 | 11.5 | 13.9 | 1.91 | 1.91 | 3.07 | 3.98 | 6.58 | 11.6 | 59.6 | 1.00 | 3.13 | 6.13 | 28.9 | 310.6 | 467.5 |
| 13 | 0.81 | 9.0 | 19.8 | 1.67 | 1.67 | 2.62 | 3.40 | 5.57 | 13.4 | 43.5 | 1.00 | 2.56 | 4.96 | 21.5 | 234.1 | 363.9 |
| 14 | 0.76 | 10.5 | 15.0 | 1.81 | 1.82 | 2.90 | 3.76 | 6.19 | 11.9 | 53.4 | 1.00 | 2.91 | 5.68 | 26.0 | 281.3 | 428.0 |
| 15 | 0.76 | 10.4 | 15.2 | 1.80 | 1.80 | 2.88 | 3.73 | 6.14 | 12.0 | 52.6 | 1.00 | 2.88 | 5.62 | 25.7 | 277.4 | 422.7 |
| 16 | 0.75 | 10.7 | 14.7 | 1.83 | 1.83 | 2.93 | 3.79 | 6.26 | 11.8 | 54.5 | 1.00 | 2.95 | 5.76 | 26.5 | 286.3 | 434.8 |
| 17 | 0.75 | 10.7 | 14.8 | 1.83 | 1.83 | 2.92 | 3.79 | 6.25 | 11.9 | 54.3 | 1.00 | 2.94 | 5.74 | 26.4 | 285.3 | 433.4 |
| 18 | 0.76 | 10.5 | 15.1 | 1.81 | 1.81 | 2.89 | 3.74 | 6.17 | 12.0 | 53.0 | 1.00 | 2.89 | 5.65 | 25.8 | 279.4 | 425.4 |
| 19 | 0.77 | 10.0 | 15.9 | 1.77 | 1.77 | 2.81 | 3.64 | 5.99 | 12.2 | 50.2 | 1.00 | 2.80 | 5.46 | 24.6 | 266.4 | 407.7 |
| Mean | | 11.9 | 14.1 | 1.95 | 1.95 | 3.14 | 4.06 | 6.74 | 11.7 | 62.1 | 1.00 | 3.21 | 6.30 | 30.0 | 322.0 | 482.9 |
| Std. Dev. | | 1.38 | 1.67 | 0.13 | 0.14 | 0.25 | 0.32 | 0.56 | 0.46 | 8.93 | 0.00 | 0.31 | 0.64 | 4.09 | 41.91 | 56.6 |
| C.V. | | 11.6 | 11.8 | 6.89 | 7.04 | 8.05 | 7.78 | 8.29 | 3.94 | 14.4 | 0.00 | 9.78 | 10.1 | 13.6 | 13.01 | 11.7 |
| Std. Dev. of mean | | 0.32 | 0.38 | 0.03 | 0.03 | 0.06 | 0.07 | 0.13 | 0.11 | 2.05 | 0.00 | 0.07 | 0.15 | 0.94 | 9.62 | 13.0 |
| C.V. of mean | | 2.67 | 2.70 | 1.58 | 1.61 | 1.85 | 1.78 | 1.90 | 0.90 | 3.30 | 0.00 | 2.24 | 2.32 | 3.13 | 2.99 | 2.69 |

*MCA: methylcarbazole; DMCA: dimethylcarbazole; Std. Dev.: standard deviation; Std. Dev. of mean = standard deviation /$\sqrt{n}$, n=19; C.V.: coefficient of variation = standard deviation / mean×100 (%); C.V. of mean: Std. Dev. of mean / mean×100 (%). $\lambda_{f}/\lambda$ is dimensionless.

Table S4 SMFI_λ_s of the alkylcarbazoles for the studied petroleum samples from the Xifeng Oilfield*.

| No. | Wells | 1-  MCA | 4-  MCA | 1,8-  DMCA | 1,4-  DMCA | 1,5-  DMCA | 1,3-  DMCA | 1,6-  DMCA | 1,2-  DMCA | 3,4-  DMCA | 1,7-  DMCA | 2,4-  DMCA | 2,5-  DMCA | 2,7-  DMCA | 2,3-  DMCA | 2,6-  DMCA |
| --- | --- | --- | --- | --- | --- | --- | --- | --- | --- | --- | --- | --- | --- | --- | --- | --- |
| 1 | X130 | 63.8 | 48.5 | 94.0 | 93.8 | 87.8 | 86.0 | 74.8 | 55.3 | 63.8 | 48.5 | 87.1 | 73.9 | 20.8 | 7.02E-07 | 1.16E-12 |
| 2 | D68-54 | 63.3 | 53.3 | 94.8 | 94.6 | 89.8 | 87.9 | 78.6 | 62.8 | 63.3 | 53.3 | 89.3 | 78.5 | 28.6 | 1.11E-06 | 1.93E-09 |
| 3 | D58-70 | 60.8 | 50.6 | 94.5 | 94.5 | 88.9 | 87.3 | 77.2 | 60.6 | 60.8 | 50.6 | 87.9 | 76.8 | 25.0 | 1.33E-06 | 2.32E-10 |
| 4 | X44-039 | 63.4 | 53.0 | 94.8 | 94.7 | 89.8 | 87.8 | 78.7 | 62.7 | 63.4 | 53.0 | 88.8 | 78.3 | 28.4 | 1.88E-06 | 7.51E-09 |
| 5 | X161 | 60.6 | 51.3 | 94.4 | 94.3 | 88.9 | 87.3 | 77.1 | 60.1 | 60.6 | 51.3 | 88.3 | 76.5 | 25.8 | 7.01E-07 | 3.25E-10 |
| 6 | X110 | 59.8 | 49.3 | 94.4 | 94.4 | 88.9 | 86.8 | 76.4 | 58.5 | 59.8 | 49.3 | 87.6 | 75.5 | 24.3 | 5.98E-06 | 1.22E-11 |
| 7 | X33-26 | 73.3 | 59.3 | 96.5 | 96.1 | 92.4 | 91.3 | 84.3 | 71.3 | 73.3 | 59.3 | 92.0 | 83.6 | 38.1 | 2.78E-05 | 4.23E-07 |
| 8 | X34-023 | 67.6 | 56.0 | 95.5 | 95.3 | 90.8 | 89.5 | 80.9 | 65.6 | 67.6 | 56.0 | 90.0 | 80.1 | 30.6 | 9.90E-06 | 2.31E-08 |
| 9 | X28-09 | 69.4 | 58.0 | 94.6 | 94.4 | 90.2 | 88.5 | 81.5 | 67.8 | 69.4 | 58.0 | 89.7 | 80.9 | 42.1 | 3.66E-04 | 7.21E-06 |
| 10 | X167 | 70.0 | 59.9 | 96.1 | 95.9 | 91.9 | 90.6 | 82.8 | 67.9 | 70.0 | 59.9 | 91.2 | 81.8 | 36.8 | 2.50E-04 | 3.25E-07 |
| 11 | X28-8 | 71.6 | 62.3 | 95.9 | 95.9 | 92.0 | 90.8 | 83.7 | 70.6 | 71.6 | 62.3 | 92.0 | 83.9 | 39.1 | 1.33E-03 | 4.98E-06 |
| 12 | X27-17 | 70.7 | 63.0 | 96.3 | 96.2 | 92.3 | 91.2 | 83.2 | 69.6 | 70.7 | 63.0 | 91.8 | 83.3 | 36.6 | 1.59E-04 | 1.84E-07 |
| 13 | X27-23 | 71.8 | 63.5 | 96.3 | 96.2 | 92.5 | 91.3 | 84.4 | 71.5 | 71.8 | 63.5 | 92.1 | 84.0 | 40.3 | 1.63E-04 | 5.10E-07 |
| 14 | X26-28 | 73.9 | 65.2 | 96.4 | 96.3 | 92.9 | 91.9 | 85.7 | 73.3 | 73.9 | 65.2 | 92.9 | 85.6 | 44.0 | 5.10E-03 | 2.05E-05 |
| 15 | X21-25 | 72.7 | 64.1 | 96.4 | 96.3 | 92.9 | 91.4 | 84.4 | 71.5 | 72.7 | 64.1 | 91.9 | 83.7 | 39.3 | 3.33E-04 | 7.78E-07 |
| 16 | X30-34 | 74.6 | 65.4 | 96.6 | 96.6 | 92.6 | 91.8 | 84.7 | 72.2 | 74.6 | 65.4 | 92.0 | 84.0 | 40.5 | 7.78E-04 | 1.68E-06 |
| 17 | X33-39 | 74.0 | 64.5 | 96.2 | 96.2 | 92.6 | 91.1 | 83.8 | 70.8 | 74.0 | 64.5 | 91.9 | 83.3 | 39.7 | 1.26E-03 | 1.27E-06 |
| 18 | X27-35 | 74.4 | 65.1 | 96.7 | 96.5 | 93.1 | 91.7 | 84.7 | 72.8 | 74.4 | 65.1 | 92.3 | 84.0 | 41.1 | 2.33E-04 | 1.10E-06 |
| 19 | X29-43 | 77.8 | 67.5 | 96.8 | 96.6 | 93.3 | 92.3 | 85.9 | 73.6 | 77.8 | 67.5 | 93.0 | 85.7 | 45.1 | 1.89E-03 | 1.78E-05 |
| ∆ln(*SMFI_λ_*) | | 1.61 | 1.37 | 0.21 | 0.18 | 0.33 | 0.41 | 0.77 | 9.40 | 0.26 | 1.61 | 0.41 | 0.85 | 4.37 | 44.51 | 110.18 |

* *SMFI_λ_*: Amended Secondary Migration Fractionation Indices; ${SMFI}_{\lambda}={SMFI}^{(\lambda_{f}/\lambda)/\bar{\lambda_{f}/\lambda}}$; the unit of *SMFI_λ_* is %. MCA: methyl-carbazole; DMCA: dimethyl-carbazole; ∆ln(*SMFI_λ_*) is multiplied by 100%.

Table S5 Calculation results of non-linear regression analyses and correction factor of benzocarbazoles in the studied petroleum samples in the Rimbey-Meadowbrook reef trend of the central Alberta*.

| Benzo-  carbazoles | $lnC=ln[a_{1}(1+a_{2}Ro+a_{4}{Ro}^{2})]+a_{3}x$ | | | | | $F_{c}$ | $\lambda_{f}/\lambda$ | $a_{\lambda}$ | $K_{r}$ |
| --- | --- | --- | --- | --- | --- | --- | --- | --- | --- |
|  | $a_{1}$ | $a_{2}$ | $a_{4}$ | $a_{3}$ | r |  |  |  |  |
| BCA[a] | 0.210 | 0.003 | 206.6 | -0.0049 | 0.866 | 0.997 | 0.996 | -0.0049 | 100% |
| BCA[c] | 0.153 | 0.550 | 167.9 | -0.0039 | 0.833 | 0.945 | 1.000 | -0.0039 | 0.0% |

*The unit of $a_{1}$ is μg/g; $a_{3}$ and $a_{\lambda}$, km^-1^, $a_{\lambda}=a_{3}\cdot\lambda_{f}/\lambda$ (equation (S4)); $a_{2}$ and $a_{4}$ are dimensionless; r represents the correlation coefficient; $F_{c}$ is the correction factor for ${SMFI}_{\lambda}$ computation; $K_{r}$ is the relative sorption coefficient (%). The original data for the calculation are listed in Table S6 in Zhang et al. (2013^)5^.

**References (1-34 in the main text)**

1. Bennett, B., Larter, S.R. Partition behaviour of alkyl-phenols in crude oil/brine systems under subsurface conditions. *Geochim. Cosmochim. Acta* **61**, 4393-4402 (1997).
2. Dyreborg, S., Arvin, E., Broholm, K. Biodegradation of NSO-compounds under different redox-conditions. *J. Contaminant Hydrology* **25**, 177-197 (1997).
3. Lu, H., Peng, P.-A., Xu, X.-Y., Zhang, L.-Y. Preliminary study on the severely biodegraded crude oil in Jiyang Depression, China. *Acta Sedimentologica Sinica* **22**(4), 694-699 (2004).
4. Bao, J.-P., Zhu, C.-S. Influence of petroleum biodegradation on aromatic hydrocarbon composition and maturity indices. *Science in China (D)* **38** (Suppl. II), 55-63 (2008).
5. Li, S.-M., Zhang, B.-S., Zhang, H.-Z., Sheng, S.-Z., Zhao, M. Geochemical characteristics and affecting factors of the unusually high dibenzothiophene oils in the Tazhong Uplift. *Geoscience* **25**(6), 1108-1120 (2011).
6. Peters, K.E., Walters, C.C., Moldowan, J.M. *The Biomarker Guide* 176-230 (Cambridge University Press, 2005).
7. Larter, S.R. et al. Reservoir geochemistry: a link between reservoir geology and engineering? *SPE Reser. Eng.* **12**(1), 12-17 (1997).
8. Hwang, R.J., Heidrick, T., Mertani, B.Q., Li, M. Correlation and migration studies of North Central Sumatra oils. *Org. Geochem.* **33**. 1361-1379 (2002).
9. Wang, T.G., He, F., Li, M., et al. Alkyl-dibenzothiophenes: Molecular tracers for filling pathway in oil reservoirs. *Chinese Science Bulletin* **49**(22), 2399-2404 (2004).
10. Qian, H., Ma, Z. *Hydrologic Geochemistry* 97-99 (Geological Publishing House, Beijing, 2005).
11. Li, R., Li, H., Xu, C., Liu, X., Tian, R., Zhu, H., Wu, L. Analytical models for describing cation adsorption/desorption kinetics as considering the electrostatic field from surface charges of particles. *Colloids and Surfaces A: Physicochem. Eng. Aspects* **392**, 55-66 (2011).
12. Dorban, M., et al. Identification of carbazoles and benzo- carbazoles in a coker gas of oil and influence of catalytic hydrotreatment on their distribution. *Fuel* **63**, 565-570 (1984).
13. Bakel, A.J., Philp, R.P. The distribution and quantitation of organitrogen compounds in crude oils and rock pyrolysates. *Org. Geochem.* **16**, 233-367 (1990).
14. Larter, S.R. Reservoir geochemistry as a reservoir appraisal and management tool, an evaluation. *AAPG Bulletin* **79**(8), 27-28 (1995).
15. Lawrence, S.R., Cornford, C. Basin geofluids. *Basin Research* **7**(1), 1-7 (1995).
16. Šráček, O., Zachariáš, J. Fluids in Geological Processes. *Earth System: History and Natural Variability Vol. 2, (*eds Cilek, V. and Smith, R.H.) 1-5 (EOLSS Publishers Company, 2009).
17. Yardley, B.W.D., Bodnar, R.J. Fluids in the Continental Crust. *Geochemical Perspectives* **3**(1), 1-98 (2014).
18. Mumm, A.S., Brugger, J., Zhao, C., Schacht, U. Fluids in geological processes — The present state and future outlook. *J. Geochem. Explor.* **106**, 1-7 (2010).
19. Mansoori, G.A. Modeling of asphaltene and other heavy organic depositions. *J. Petrol. Sci. Eng.* **17**, 101-111 (1997).
